# Different levels of statistical learning - Hidden potentials of sequence learning tasks

**DOI:** 10.1371/journal.pone.0221966

**Published:** 2019-09-19

**Authors:** Emese Szegedi-Hallgató, Karolina Janacsek, Dezso Nemeth

**Affiliations:** 1 Doctoral School of Psychology, ELTE Eötvös Loránd University, Budapest, Hungary; 2 Institute of Psychology, Faculty of Humanities, University of Szeged, Szeged, Hungary; 3 Prevention of Mental Illnesses Interdisciplinary Research Group, University of Szeged, Szeged, Hungary; 4 Institute of Psychology, ELTE Eötvös Loránd University, Budapest, Hungary; 5 Brain, Memory and Language Research Group, Institute of Cognitive Neuroscience and Psychology, Research Centre for Natural Sciences, Hungarian Academy of Sciences, Budapest, Hungary; 6 Lyon Neuroscience Research Center, Université de Lyon, Lyon, France; Texas Tech University, UNITED STATES

## Abstract

In this paper, we reexamined the typical analysis methods of a visuomotor sequence learning task, namely the ASRT task (J. H. Howard & Howard, 1997). We pointed out that the current analysis of data could be improved by paying more attention to pre-existing biases (i.e. by eliminating artifacts by using new filters) and by introducing a new data grouping that is more in line with the task’s inherent statistical structure. These suggestions result in more types of learning scores that can be quantified and also in purer measures. Importantly, the filtering method proposed in this paper also results in higher individual variability, possibly indicating that it had been masked previously with the usual methods. The implications of our findings relate to other sequence learning tasks as well, and opens up opportunities to study different types of implicit learning phenomena.

## Introduction

When previous experiences facilitate performance even though the current task does not require conscious or intentional recollection of those experiences, implicit memory is revealed [1]. The Serial Reaction Time (SRT) task [2] is a commonly used task measuring implicit learning and memory in the visuomotor domain; people are instructed to respond to a sequence of stimuli by pressing a corresponding button (usually having a 1:1 stimulus-response mapping), and even though they are not aware that the same pattern of successive trials is repeated over and over again, they nevertheless show improvement compared to their reactions to random (or pseudorandom) streams of stimuli. A drawback of the design is that learning can only be assessed at certain points (via inserting blocks of random stimuli), and that, due to the simplicity of the SRT sequences, people may become aware of them after all, in which case explicit memory is being measured instead of or in addition to implicit learning[3].

A modified version of the task, namely the Alternating Serial Reaction Time task (ASRT), has been introduced twenty years ago as a possible solution to the aforementioned problems [4]; and it turned out that even the test-retest reliability is better using this variant [5]. At the time of writing these lines, that article introducing the ASRT task had been cited three hundred times (317, to be precise), and *Google Scholar* has about 200 results for the expression „alternating serial reaction time”, out of which 87 has been published since 2015. Clearly, the task gained popularity as a research tool recently, which is not surprising given its advantages over the classical SRT. Having a lot of experience with it ourselves, we began to feel there is even more to it than currently recognized. In the present paper, we aim to discuss the potential challenges of its currently used analysis methods and to provide some ideas about how to overcome these flaws. Importantly, most of the concerns (and solutions) discussed in this paper are directly applicable to other sequence learning tasks as well.

### About the ASRT task

In the ASRT–to make the predetermined sequence less apparent—a four element long pattern (e.g. 1-4-2-3) is intervened by random elements (i.e. 1-R-4-R-2-R-3-R). Participants generally don’t recognize the pattern (or the fact that there is a pattern), and still react to pattern trials faster and more accurately than to random trials (referred to as *pattern learning*). Moreover, the relative advantage of pattern trials can be assessed at any point of learning, or continuously throughout learning.

At first, the typical result may seem like evidence that people are capable of somehow detecting the pattern–and thus being able to respond to these elements more efficiently–even though pattern trials are hidden between random elements), but this is not necessarily the case. As a consequence of the alternation of pattern and random trials, some stimulus combinations are more frequent than others and some trials are more predictable than others, possibly leading to faster and more accurate responses to them (referred to as statistical learning). When assessing stimulus combinations of at least three consecutive trials, the variability of such combinations depends on the number of random elements they contain. For example, random-ending triplets (three consecutive trials, i.e. R-P-R) are four times as variable as pattern-ending triplets (i.e. P-R-P), since they contain two random elements instead of one. Accordingly, particular R-P-R combinations occur with a much lower frequency than particular P-R-P combinations. Moreover, some of the R-P-R triplets mimic P-R-P triplets (e.g. 1-2-2 can occur as both an R-P-R and a P-R-P triplet) further increasing the frequency of those instances, and further increasing the difference between the so-called „high-frequency triplets” and „low-frequency triplets”. What’s important is that most of the high-frequency combinations end on pattern trials, while all of the low-frequency combinations end on random trials, and this way trial type (pattern vs. random) and statistical features of stimuli are heavily confounded. Not surprisingly then, learning can be detected by contrasting trial types or by contrasting triplet types (irrespective of which of the two information types drive learning). Both methods can be found in the literature: some researchers treat the ASRT as a pattern-learning task, which is revealed by making comparisons solely on the basis of trial type (pattern vs. random), e.g. [6–9]. Others treat the task as a statistical learning task, since their comparisons are being made solely on the basis of triplet type (frequent vs. infrequent) while ignoring trial type (pattern vs. random), e.g. [10–15,5]. Finally, in the minority of cases, both factors (trial type and triplet type) are considered simultaneously, e.g. [4,16,17,17–20], making it possible to assess the relative contribution of the two learning types.

Howard and Howard [4], for example, compared high-frequency triplets that end on random trials (hereinafter RH), high-frequency triplets that end on pattern trials (hereinafter PH) and low-frequency triplets always ending on random trials (hereinafter RL). They found that RH trials were responded to faster and more accurately than RL trials—thus triplet frequency learning did occur. This result couldn’t be attributed to pattern learning since only responses to random trials were compared. At the same time, they also found that PH trials were reacted to faster and more accurately than RH trials, possibly indicating pattern (rule) learning. As a reminder, these trials are the ending trials of the same triplets (e.g. 1-2-**2**), but one of the triplets is an R-P-R triplet (thus the critical, final trial is a random trial) while the other is a P-R-P triplet (thus the final trial being a pattern trial). But here is the catch: although triplet level statistical information couldn’t act as a confound in this measure, higher order statistical information could (i.e. although the trials being compared are the ending trials of identical triplets, they differ on the N-3^th^ trial). The authors, recognizing this, used the term *higher order learning* when referring to the measure derived from contrasting RH and PH trials.

From Howard & Howard’s [4] work we do know now that triplet level statistical learning occurs in the task, but we still don’t know whether it’s pattern learning and/or higher order statistical learning that explains improvement of performance that cannot be attributed to triplet level statistical learning (i. e. *higher order learning*). We only know that there is a little *extra* to triplet learning. And albeit being little, this extra is not marginal; this measure differentiates between age groups [4]. From modified versions of the ASRT task we also know that higher order *statistical* learning is possible: it has been shown that even third-order statistical regularities can be learned by humans, and also that such learning is reduced in the old compared to the young [21,22], just as the *higher order learning* measure is reduced in elderly. But, of course, this does not exclude the possibility that pattern learning also occurs in the ASRT.

Despite the uncertainty that remains about this measure, it is still surprising that only a handful of studies quantified it at all, as it costs nothing to do so and it opens new opportunities for data interpretation. First, if overall differences exist between groups, it can be determined whether differences arise from triplet level learning, higher order learning or both; and second, when no overall differences are detected with the simpler methods, it may be due to decreased sensitivity to detect higher order (subtler) learning. Indeed, only a few studies reported group differences in the ASRT literature using the less elaborate analysis methods, e.g. [16,23–27,13,28–32]. We need to increase the sensitivity of the employed analysis methods in order to make the ASRT a truly effective tool measuring implicit learning capabilities. One way of doing so is differentiating between different kinds and levels of learning that can be detected, and that are confounded in the typical analyses. Not only would this result in more diverse information about a particular participant’s learning ability, but also in purer measures. The ASRT task might be a goldmine, we should stop digging coal.

### Statistical properties and analysis methods of the task

We have talked about how pattern learning is confounded by statistical learning, and how unclear it is what constitutes „higher order learning”. The story is however even more complex. For example, in the typical analyses of ASRT data no distinction is being made between joint probability learning (how frequent a particular combination is, e.g. 1-2-2) and conditional probability learning (how often does 1-2-… end with 2), and although the terminology points to the former (e.g. the terms „low-frequency triplet” and „high-frequency triplet”), the way we typically analyze data is more in line with the latter (since we analyze reaction times given to the final elements of triplets; i.e. we measure whether a particular response is faster following a specific set of trials contrasted with different sets of trials). Humans are capable of both kinds of statistical learning [33], and as we will show, the ASRT task has the potential to distinguish between the two. Furthermore, pattern learning and higher than second-order statistical learning can also be separated (even if not perfectly, but at least to a higher degree than we used to).

**Fig 1:**
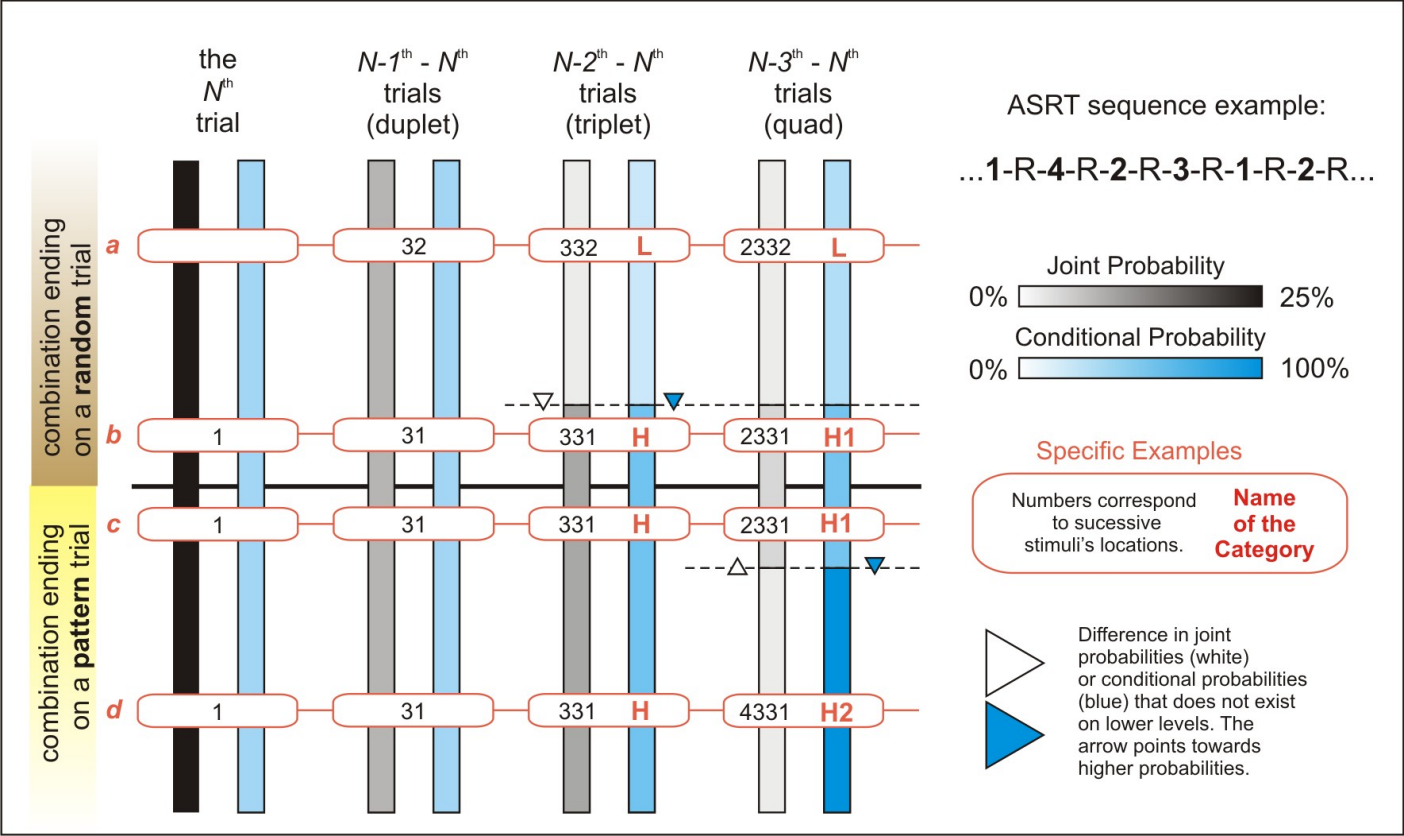
Statistical properties of the ASRT trials and trial combinations. Shades of gray represent combination frequencies. Shades of blue represent the predictability of a given trial. Darker shades represent higher frequencies/probabilities. Zero to three preceding trials are taken into consideration–see clusters of bars from left to right). Each bar represents the total number of trials/combinations on a given level (e.g. one-third of a bar represents one-third of the combinations on that level). The upper half of the bars represent combinations that end on a random trial, while the lower half represents combinations that end on a pattern trial. The points of the bars that are at the same height represent the same trials (considering 0–3 antecedent trials when moving from left two right); connected boxes show specific examples of the categories.

We summarized trial probabilities and combination frequencies on Fig 1 using two color scales (shades of gray representing combinations frequencies and shades of blue representing the predictability of a given trial; darker shades represent higher frequencies/probabilities) on four levels (0–3 preceding trials taken into consideration). Each bar represents the total number of trials/combinations on a given level (e.g. one-third of a bar represents one-third of the combinations on that level). The upper half of the bars represent combinations that end on a random trial, while the lower half represents combinations that end on a pattern trial. The points of the bars that are at the same height represent the same trials (considering 0–3 antecedent trials when moving from left two right); four examples are shown in boxes connected via red lines.

Several things may be noticed by looking at Fig 1:

1. Single trials (1, 2, 3 or 4) or duplets (e.g. 12, 13, 14, etc.) are of uniform statistical properties throughout the sequence since the first two groups of bars are of uniform color. In 50% of the cases, these trials/duplets end on pattern trials (bottom halves of the bars), in the remaining cases the same combinations end on random trials (top halves of the bars; contrast the examples *b* and *c*, for example, showing that the combination 31 occurs both ways)
2. When at least two preceding trials are taken into consideration (triplets, quads, etc.) some trials are more predictable than others (blue bars are not uniformly colored) and some combinations are more frequent than others (gray bars are also not uniformly colored either). Moreover, these categories do not overlap perfectly, as, for example, when considering quads, there are only two different shades of gray but three shades of blue (meaning two categories on the basis of combination frequencies, and three categories on the basis of conditional probabilities). Higher joint probabilities sometimes correspond to higher conditional probabilities, e.g. when considering triplets; other times they go in different directions, e.g. when considering quads.
3. On the level of triplets, two categories can be distinguished based on joint probabilities, and the same category boundaries separate trials with different conditional probabilities. E.g. the combination 332 is less frequent than the combination 331 (light gray vs. darker gray part of the first bar), and simultaneously, after the preceding trials 33 it is more probable that a stimulus 1 will follow and not the stimulus 2 (light blue vs. dark blue part of the second bar). The category with the higher probabilities (both joint and conditional) is denoted as H, while the category with the lower probabilities is denoted as L, see the examples *a* vs. *[b and c and d]* on Fig 1.

Members of the H category can further be divided into two subcategories when the N-3^th^ trial is considered (the new categories being H1 and H2 quads, respectively). With the usual analysis methods, there was no distinction being made between these two quad types. As it can be read from the figure, H1 quads are more frequent than H2 quads (see the dark vs. light gray colors of the first bar), but the final trial of H1 combinations is less probable given its antecedents than the final trial of H2 combinations (see the light blue vs. darker blue colors of the second bar). So, for example, 2331 is a more frequent combination than 4331, but while combinations starting with 433 consistently end with 1, combinations starting with 233 can end with 1, 2, 3 or 4.

As noted earlier, some researchers analyze the data gathered with the ASRT task by contrasting trials of different *Trial Type*, i.e. pattern and random trials, e.g. [6–9], resulting in a learning measure called *Pattern Learning* or *Trial Type Effect*. On Fig 1 this corresponds to contrasting the upper half of the trials/combinations with the lower half, i.e. contrasting the exemplars *a*, *b* with *c*, *d*. This kind of analysis bears on the implicit assumption that the ASRT is primarily a rule-learning (pattern-learning) task, and does not take statistical properties into consideration, albeit being heavily confounded by them; e.g. when considering triplets, members of the H category (e.g. 331, examples *c*, *d*) are contrasted with a mix of H and L category members (331 and 332, examples *a*, *b*). We will refer to this analysis method as Model 1.

Other times the assumption is that the ASRT is primarily a triplet learning task (thus a statistical learning task). The learning measure is derived from contrasting performance on H vs. L category members resulting in a measure called *sequence-specific learning*, *sequence learning effect* or *triplet type effect*, e.g. [34,10–15,5]. This model does not explicitly deal with the possibility of higher-order learning (e.g. quad level and higher), and thus it does not differentiate between combination frequency learning and trial probability learning (since the correlation between the two is 100% up to the level of triplets). It also doesn’t take Trial Type (pattern vs. random) into consideration, albeit these factors are confounded; e.g. 332 only occurs as a combination ending on a random trial (example *a* on Fig 1), but 331 occurs both ways (examples *b*, *c*, *d* on Fig 1). Hereinafter we will refer to this analysis method as Model 2.

A third analysis tradition considers both triplet level statistical information,(i.e. Triplet Type; H and L categories) and Trial Type (pattern vs. random trials), e.g. [4,16,17,17–19]. This model distinguishes three categories and three learning measures: the difference between performance on random-ending L (LR)and random-ending H (HR) trials is usually called *pure statistical learning* (examples *a* vs. *b* on Fig 1), the difference between random-ending H (HR) and pattern-ending H (HP) trials is called *higher order sequence learning* (examples *b* vs. *[c* and *d]* on Fig 1), while the difference between LR and HP trials is called *maximized learning* [17] (examples *a* vs. *d* on Fig 1). Hereinafter we will refer to this analysis method as Model 3. Importantly, this method treats pattern trials as a uniform category, while in reality pattern trials can be divided into the subcategories H1 and H2 considering quad level statistical information (e.g. high frequency triplets such as 331 may be part of quads 2331 –H1 category–or 1331 / 3331 / 4331 –H2 category, see Fig 1 examples *b*, *c* and *d*). This is particularly important as the *Higher Order Learning* measure was the one to differentiate between age groups [4], and the authors raised the possibility themselves that the measure might include higher level statistical learning in addition to or instead of pattern learning. The problem is that the Higher Order Sequence Learning measure contrasts quads from the H1 statistical category with quads from both H1 and H2 categories. If the driving force of learning is indeed statistical information, it is plausible to assume that this measure is underestimated, as the difference between H1 and (H1+H2) quads must be smaller than the difference between pure groups of H1 and H2 quads. Thus, we suggest that „higher order statistical learning” (i.e. quad learning) could be detected more efficiently if H1 quads would be contrasted with H2 quads instead of contrasting HP and HR trials (i.e. we suggest contrasting *a* vs. *[b and c]* vs. *d* instead of contrasting *a* vs. *b* vs. *[c and d]* on Fig 1).

For this reason, we introduce Model 4 and Model 5 in this paper as possibly better analysis methods. In Model 4, quad level statistical information is considered, but Trial Type (random vs. pattern) is not. Thus, it treats the ASRT as a solely statistical learning task with no rule-learning (pattern-learning) component. The categories being compared are L, H1 and H2 (*a* vs. *[b and c]* vs. *d* on Fig 1, quad columns). Importantly, trial predictability (i.e. conditional probabilities) and combination frequencies dissociate clearly in this case: H2 combinations are less frequent than H1 combinations but their final trial can be anticipated with much higher probability given the first three trials of the combination The difference between L and H1 trials could be called *triplet learning* (*+ pattern learning*), the difference between H1 and H2 trials *quad learning* (*+ pattern learning*), while the difference between L and H2 trials could be called *maximized learning*. In this Model, the categories differ more clearly in the statistical aspect and less clearly in *Trial Type*, while the opposite was true in Model 3 (hence the parentheses in the suggested names for the learning measures; trial type learning is secondary in Model 4). Thus, if the primary driving force of learning is sensitivity to statistical information (rather then sensitivity to the hidden pattern), Model 4 should fare better than Model 3, and vice versa.

Lastly, Model 5 would consider both *Trial Type* (pattern or random) and *Quad Type* (L, H1 and H2 categories), resulting in four categories (examples *a* vs. *b* vs. *c* vs. *d* on Fig 1, quad columns, considering whether the combination ends on a pattern or random trial as well). Again, on the level of quads, trial predictability and combination frequency dissociate (they point in the opposite direction), thus their relative impact might be assessed just as with Model 4. As a bonus, random-ending H1 trials (H1R) can be contrasted with pattern-ending H1 trials (H1P), leading to a pattern-learning measure that is less confounded by statistical information than the pattern-learning measures of the previous models. We propose the following names for the resulting learning measures: *triplet learning* (LR vs. H1R; examples *a* vs. *b* on Fig 1), *pattern learning* (H1R vs. H1P; examples *b* vs. *c* on Fig 1), *quad learning* (H1P vs H2P; examples *c* vs. *d* on Fig 1) and *maximized learning* (LR vs. H2P; examples *a* vs. *d* on Fig 1).

**Fig 2:**
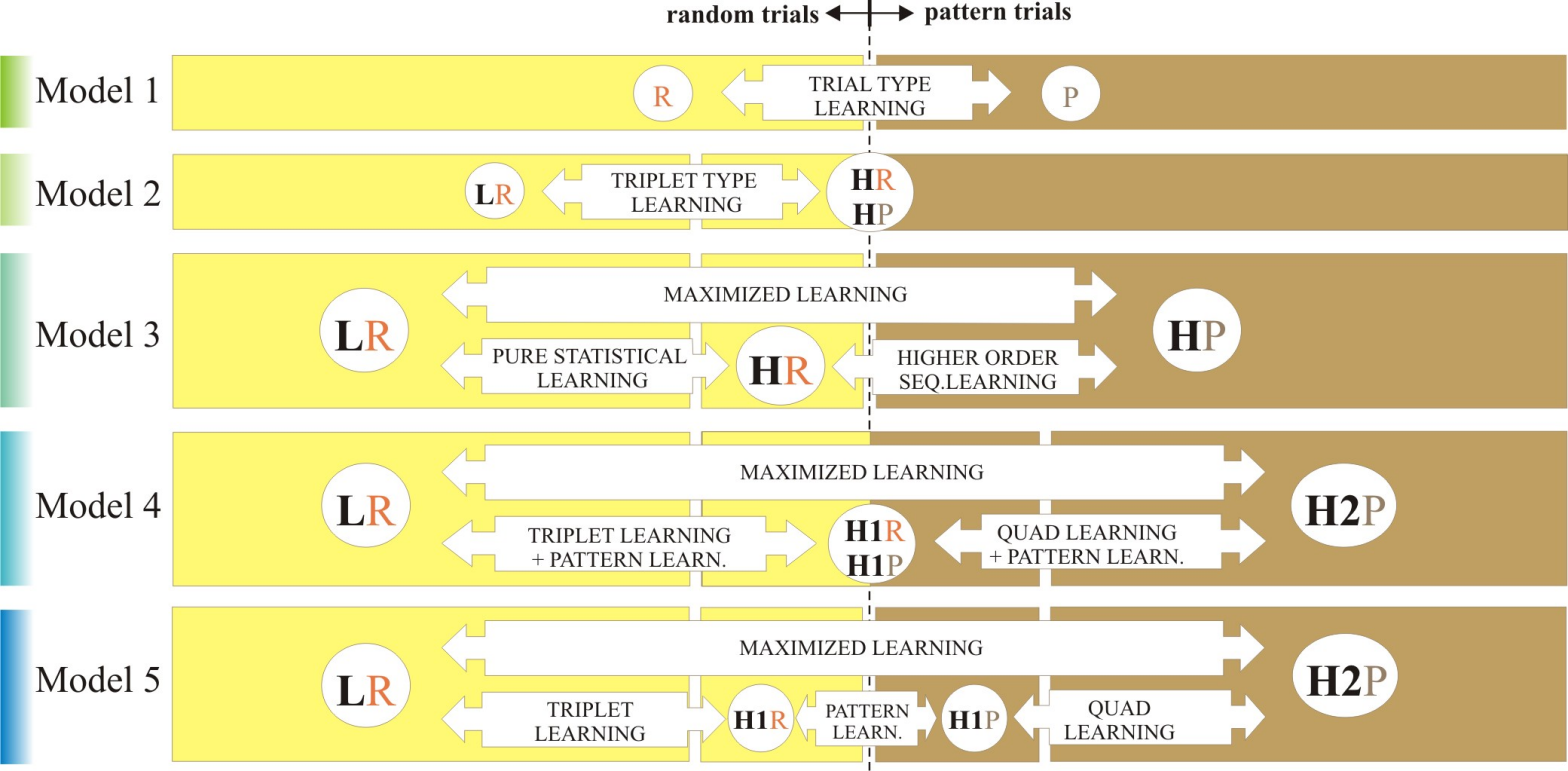
Different models of the ASRT task as a basis of extracting different learning scores. P–pattern trials, R–random trials, L–low probability trials, H–high probability trials (H1 and H2 being subcategories of the latter; H2 trials are more probable than H1 trials, but at the same time triplets that end on a H2 trial are less frequent than triplets that end on a H1 trial). Models 1–3 has been typically used as a basis of data analysis; Model 4–5 are introduced in this paper.

An overview of Model 1 to Model 5 is illustrated on Fig 2. One of the aims of the current study was to compare these models in terms of goodness of fit, thus to decide whether it pays off to use a more elaborate model when analyzing ASRT data.

### Confounding variables in the ASRT task

The ASRT task is a reaction time task, and reaction times vary as a function of many factors. Fatigue (its effects could be approached as in [35]), boredom, stimulus timing, the number of response locations etc. may all affect the magnitude and variability of reaction times [36–38], and thus our ability to detect learning on the task, or even learning itself. But these factors at least have a similar impact on the groups of trials that are to be contrasted in the ASRT task—either because they are constant (e.g. response-stimulus interval), or because the different trial types are evenly distributed throughout the task, thus even time-dependent factors such as fatigue have similar effects on the different trial types in a given time window.

There is, however, at least one factor which may act as a confounding variable: for some stimulus combinations, e.g. serial repetitions of the same stimuli, response facilitation is observed when contrasted with other combinations, e.g. an inconsistent pattern of alternations and repetitions. These so-called *sequential effects* [39] are evincible in random streams of stimuli, but also from reaction time tasks in which the conditional probabilities of stimuli vary, see [40]. We have no solid idea of exactly which combinations should be relatively „easier” (facilitated) compared to others because this phenomenon has mostly been studied in binary-choice reaction time tasks, e.g. [41–43] (but see [44]), those combinations being less numerous and less complex than the combinations in the ASRT task. Also, the type and direction of these effects depend strongly on the response to stimulus interval (RSI). The automatic facilitation effect (that is of interest to us) typically occur with RSIs of 100 ms or less, although the exact values tested vary from experiment to experiment, as summarized by [45], and these results are mostly derived from two-choice reaction time tasks and thus might not apply for the ASRT. In the absence of concrete expectations of how and to what extent sequential effects occur in the ASRT (and bearing in mind that ASRTs with different RSIs may differ in this regard), the wisest thing we can do is to ensure that the groups of trials that are to be contrasted in the ASRT (e.g. pattern vs. random trials or highly predictable vs. moderately/slightly predictable trials, etc.) belong to the same types of combinations with respect to local sequential effects (“easy” or “hard”).

The most influential proposal that aimed at reducing unwanted sequential effects was of Howard et al. [21] who eliminated spans (also called *trills*, e.g. *a-b-a*) and repetitions (e.g. *a-a-a*) from the analysis since these types of triplets always occur as random trials for each participant (irrespective of the particular sequence being taught). As Song and her coworkers put it, “perfomance on trills and repetitions could reflect preexisting biases, rather than sequence learning” [46]. Each of the remaining 48 triplets can be described in the abstract form as *cba*, *bba or baa* (where different letters represent different stimuli), and the proportion of these types of triplets is similar in the groups being compared on the basis of their statistical properties (i.e. high- vs. low-frequency triplets). Even if one type of triplets, e.g. *baa* is easier than the other types of triplets (since it ends on a repetition), this shouldn’t pose a problem because the proportion of *baa* triplets is similar across high- and low-frequency triplets. Moreover, each of the 48 individual triplets have an equal chance of being a high-frequency triplet (they are high-frequency triplets in some of the sequences, and low-frequency triplets for the remaining sequences), thus, on the group level, pre-existing biases shouldn’t prevail. Since in this case filtering is applied at triplet level, hereinafter we refer to this method as *triplet filtering*.

Interestingly, Song et al. [46] found that preexisting biases can be found even on the level of quads. At the beginning of their ASRT, RH triplets were temporarily faster and more accurately reacted to than PH triplets–for a similar finiding see [17]. In order to eliminate possible preexisting biases that could cause this effect, they categorized quads into seven categories: “those that contain two repeated pairs (i.e., 1122); a repeated pair in the first (1124), second (1224), or last (1244) position; a run of three in the first position (1112); a trill in the first position (1213); or no repeated elements (1243)” (p. 170.). After removing all the unequally represented quad types of this sort, the unexpected difference between RH and PH trials in the first session disappeared, whereas the difference between low- and high-frequency triplets remained. Although the paradoxical RH-PH difference only manifested in the first session (after 150 repetitions of the pattern), and reversed afterward, it is still quite surprising that this method of eliminating pre-existing biases on the level of quads did not become commonly used. One reason might be that the description of this method was limited to a few words in a footnote.

In this work, we propose the elimination of quad level preexisting biases by using a similar method. Our notations were derived the following way: whatever the current stimulus was (position 1, 2, 3 or 4), it was denoted as *„a”*. If the previous stimulus was identical to the current one, it was also denoted as *„a”*, thus the combination of the two was denoted as *„aa”*. Otherwise, if the previous stimulus was different, the combination was denoted as *„ba”*. Going further, if the N-2^th^ trial was identical to the N-1^th^ or N^th^ trial, it was denoted with the same letter as the one that it was identical to (e.g. „aba” or „bba”); otherwise, it got the following letter from the alphabet (e.g. *„c”*). This way a quad that consistsed of four different stimuli was always denoted as *„dcba”* (irrespective of whether it was derived from 1-2-3-4, 3-1-4-2 or else). Importantly, we assigned these letters to stimuli starting with the N^th^ trial and going backward in order to be able to match combinations of different lengths. For example, a triplet consisting of three different stimuli was „**cba**”, and the same triplet could be part of a quad „a**cba**”, „b**cba**”, „c**cba**” or „d**cba**”.

As a difference to Song et al. [46], our quad categories rely on the abstract structure of the quads, thus differentiating between 1-2-2-1 and 3-2-2-1 quads (*abba* vs. *cbba*), and between 1-2-1-1 and 3-2-1-1 quads (*abaa* vs. *cbaa)*, too; moreover we observed a category that was not mentioned by Song et al. [46], namely *acba* quads (e.g. 1-2-3-1). Only three out of 13 categories are counterbalanced across the groups of trials being compared within subjects (e.g. P vs. R in Model 1, L vs. H in Model 2, etc., see Fig 2) and across participants (i.e. any particular quad having an equal chance of belonging to either statistical category). These quad types are *dcba*, *cbba* and *acba*. Hereinafter, we will refer to this filtering method as *Quad Filtering*. As a specific example of the possible benefit of using the Quad Filtering is the elimination of *bbaa* quads (e.g. 1122, 1133, 2233, etc.), which seem to be the easiest (fastest) combinations of all. These combinations only occur as members of the H1 category, moreover, they constitute approximately 25% of that category. Different repetition-ending combinations (e.g. *abaa*, *cbaa*) do occur in other statistical categories as well (e.g. L, H2), and they are also reacted to relatively fast (compared to nonrepetition-ending combinations), but they only make up 8–16% of a particular category. In other words, H1 category is, on average, easier than the L or H2 categories, which might manifest in overestimated Triplet Learning measures and either underestimated Quad Learning measures (if dominantly conditional probabilities are being learned) or overestimated Quad Learning measures (if dominantly joint probabilities are being learned). Using the previous models of ASRT, this bias could have manifested as an overestimation of the Pure Statistical Learning measure of Model 3, and either in an underestimation of the Higher Order Learning measure—given that learning is driven by conditional probabilities (it could also cause the paradoxical negative difference between the HR and HP categories), or an overestimation of the same measure (given that learning is driven by joint frequencies).

We would like to highlight, however, that these filtering methods do not necessarily eliminate pre-existing biases on the individual level. Even if the percentage of, say, *dcba* quads is counterbalanced across statistical categories (i. e. within participants) and across participants, it is still reasonable to assume that some of these quads are easier than others. For example, Lee, Beesley, & Livesey [44] observed that sequences of trials with many changes in directions (of movements) are harder to react to than sequences of trials where the direction of movement does not change (e.g. 1-4-2-3 is harder than 1-2-3-4). Since quads are not counterbalanced in this respect within participants, learning on some or all of the sequences might be confounded by such biases despite being controlled for on the group level. This is particularly important if we aim is to measure individual differences in ASRT learning: any correlation with other measures (or the lack of correlation) might be due to such confounds.

Also, while the elimination of quad level pre-existing biases on the group level should make the interpretation of the results more straightforward, one has to keep in mind that higher level sequential effects could still act as a confound. Lee et al. [44] observed that lower level sequential effects had a higher effect size than higher order sequential effects (e.g. *η*_*p*_^*2*^ = 0.881 on the triplet level vs. *η*_*p*_^*2*^ = 0.275 on the quad level and *η*_*p*_^*2*^ = 0.314 on the quint level), but the latter nevertheless influenced reaction times. This indicates that such biases should be controlled for at least on the level of quints, or on even higher levels, to minimize their impact when assessing statistical learning. Unfortunately, there are no quints that are counterbalanced across all the relevant statistical categories across participants, so there is no easy way of assessing the impact of preexisting biases on this level. Further studies should investigate the magnitude of such biases, for example by using random series of stimuli in a 4-choice (ASRT-like) reaction time tasks and assessing the preexisting tendencies when reacting to quints that also occur in the ASRT sequences.

### The aim of the study

In the previous section, we described how different types of information might be the basis of learning or might influence learning in the ASRT task, such as trial type, trial probability, combination frequency and preexisting biases to certain stimulus combinations. We also noted that there are at least three types of analysis utilized by different research groups (we will refer to these as Model 1, Model 2 and Model 3, respectively), and we made suggestions on how to improve these methods (we will elaborate on these when introducing Model 4 and Model 5 later in this text).

In the following sections, we will first review the different analysis methods (Model 1 to Model 5) with respect to possible confounds in their learning measures. We considered such a detailed description to be helpful not only for deciding which analysis method to use for one’s purposes in the future but also for the evaluation (or re-evaluation) of previous findings. Along with the description of the learning measures of these models, we will present data confirming and illustrating the theoretical considerations reviewed so far.

Second, we will compare the models (Model 1 to Model 5) in terms of goodness of fit, which might corroborate our proposals from a different perspective (i.e. how efficient a Model is in capturing the different aspects of learning) on the ASRT task. Although we will present and compare findings got with different filtering methods (No Filter, Triplet Filter, and Quad Filter), the main focus of this section will be on differences between Models (Model1-5) within Filtering Methods, and not vice versa. The reason for this is that the superiority of one filtering method over another cannot easily be justified via statistics (i. e. the purer, bias-free effect size might be smaller than the biased; it should nevertheless be preferred. Statistics can only tell us about the magnitude of effects, but not their purity).

Third, we will elaborate on the specific learning effects in each Model (Model1-Model5). These measures cannot be directly compared (remember, the reason for introducing Model4 and Model5 was the fact that specific learning measures in Model 1–3 might reflect the mixed learning of different types of information), and so the main focus of this section will be the comparison of different Filtering Methods within the Models. We will discuss the effect of these filters on the magnitude of learning and individual variability that can be detected in the task.

Last but not least, in the fourth section, we will examine the learning scores calculated with the methods that we propose. The main focus here will be on whether participants (as a group) showed learning of the different statistical properties of the sequence, and also the percentage of participants who showed learning of these properties (individually). We will also consider the time-course of learning of these aspects (e.g. does quad learning occur later in time than triplet learning?).

## Methods

We based our analysis on data ori**g**inally collected by (and published in) Török, Janacsek, Nagy, Orbán, & Németh [35] with the authors’ permission. Because of this, we copied the most relevant parts of their Methods section (not necessarily in the same order as originally provided); a more detailed description can be found in the aforementioned paper.

### Participants

One hundred and eighty healthy young adults participated in the study, mean age *M* = 24.64 (*SD* = 4.11), *Min*_*age*_ = 18, *Max*_*age*_ = 48; 28 male/152 female. All participants had normal or corrected-to-normal vision and none of them reported a history of any neurological and/or psychiatric condition. All participants provided written informed consent before enrollment and received course credits for taking part in the experiment. The study was approved by the United Ethical Review Committee for Research in Psychology (EPKEB) in Hungary (Approval number: 30/2012) and by the research ethics committee of Eötvös Loránd University, Budapest, Hungary. The study was conducted in accordance with the Declaration of Helsinki.

### Equipment

Alternating Serial Reaction Time Task The Alternating Serial Reaction Time (ASRT) task was used to measure statistical learning capabilities of individuals (J. H. Howard & Howard, 1997).

### Procedure

Participants were instructed to press a corresponding key (Z, C, B, or M on a QWERTY keyboard) as quickly and accurately as they could after the stimulus was presented. The target remained on the screen until the participant pressed the correct button. The response to stimulus interval (RSI) was 120 msec. The ASRT task consisted of 45 presentation blocks in total, with 85 stimulus presentations per block. After each of these training blocks, participants received feedback about their overall RT and accuracy for 5 seconds, and then they were given a 10-s rest before starting a new block. Each of the three sets of 15 training blocks constitutes a training session. Between training sessions, a longer (3–5 min) break was introduced.

Each participant was given a randomly chosen ASRT sequence (out of the six possible sequences). This way 32 of the participants got the sequence *1-r-2-r-3-r-4-r*, 29 participants got *1-r-2-r-4-r-3-r*, 31 participants got *1-r-3-r-2-r-4-r*, 33 participants got *1-r-3-r-4-r-2-r*, 29 participants got *1-r-4-r-2-r-3-r* and 26 participants got *1-r-4-r-3-r-2-r*.

EPRIME 2.0 was used as a stimulus presentation software [47].

### Statistical analyses

Probability distributions of continuous variables (subsets *a* vs. *b*) were compared using the *Kolmogorov-Smirnov* test and the *Mann-Whitney test*. Effect sizes for such differences were computed in the form of *Probability of Superiority*, i.e. the probability that a randomly chosen value from subset *b* is higher than a randomly chosen value from subset *a*. Distributions of nominal variables were compared using the Chi-Squared test, and Cramer’s V was computed as the corresponding effect size.

Models’ goodness of fit was computed in the form of *adjusted R-squared* values (in the case of reaction times) and *Cramer’s V* values (in the case of error data). Variability was computed in the form of *standard deviations (SD)* and *coefficients of variation (CV)*. Specific learning scores were quantified as *Cohen’s d* effect sizes (reaction times) and *Cramer’s V* values (error data). For the comparison of these values (within Models or within Filtering Methods) we used *ANOVA*s, and we reported *partial eta squared* effect sizes along with *p* values.

To assess whether the variability of two data sets is different we used the *Levene-test*. The reliability of the measures was assessed via the *split-half method*.

## Results

### Variables that contribute to the learning scores of different Models using different filtering methods

In this section we aimed to statistically confirm the considerations we discussed so far on a theoretical basis, which is not only important in order to strengthen our message, but also because the ASRT sequence is not fully pre-determined (as half of the trials are randomly determined), thus the actual sequence varies from participant to participant. While it is always true that there is 25% chance for a random stimulus to be 1, 2, 3 or 4, it is not guaranteed that in a particular sequence these outcomes will have frequencies that match their probabilities (e.g. stimulus 2 might come 32% of the time for a particular participant). According to the law of large numbers, the more trials we have, the more the actual frequencies will approach theoretical probabilities. In this study, participants performed 45 blocks of ASRT which corresponds to approximately 1750 random trials that shape the overall statistical properties of the sequence. We aimed to assess to what extent do previously described considerations apply on the individual level with this amount of random trials. Is it possible, for example, that for some participants there is a difference in actual statistical properties between two categories that should not differ based on theory (e.g. H1 and H2 trials differing in triplet level conditional probabilities)? Or is it possible that for some participants quad filtering is not effective in balancing out combination types across statistical categories? How often do these kinds of anomalies occur with the three different filtering methods?

#### Trial type proportions

As it can be read from **Fig 2**, most of the statistical categories in the different models contain only P trials (Model 1 P; Model 3 HP; Model 4 H2; Model 5 H1P and H2P) or only R trials (Model 1 R; Model 2 L; Model 3 LR and HR; Model 4 L; Model 5 LR and H1R). The only exceptions are Model 2’s H category and Model 4’s H1 category which contain both P and R trials; the H1 category is made up of 50% R and 50% P trials (regardless of the filter being used); while the H category of Model 2 consists of 20% R and 80% P trials when No Filter or Triplet Filtering is applied; and it contains 33% R and 67% P when the Quad Filter is applied (see Table A in S1 File for corresponding statistics).

Ideally only those categories should differ in the P/R proportions that are used to compute *Pattern Learning* scores, i.e. the learning that (possibly) occurs if participants are sensitive to the trial type (P or R) in addition to statistical information, such as the P vs. R category in Model 1 and the H1P vs. H1R categories of Model 5. In other cases, the differences in P/R proportions are not of a concern because the contrasts admittedly assess mixed effects of different learning types (e.g. the HP vs. HR categories of Model 3 or the Maximized Learning scores of Models 3–5). The only problematic contrasts are the L vs. H categories in Model 2 and the L vs. H1 and H1 vs. H2 categories of Model 4 (see Table A in S1 File). Remember, these models treat the ASRT as a primarily statistical learning task; nevertheless, the Triplet Learning and Quad Learning scores might be confounded by pattern learning resulting in an overestimation of statistical learning.

#### Combination frequencies (joint frequencies)

**Fig 3:**
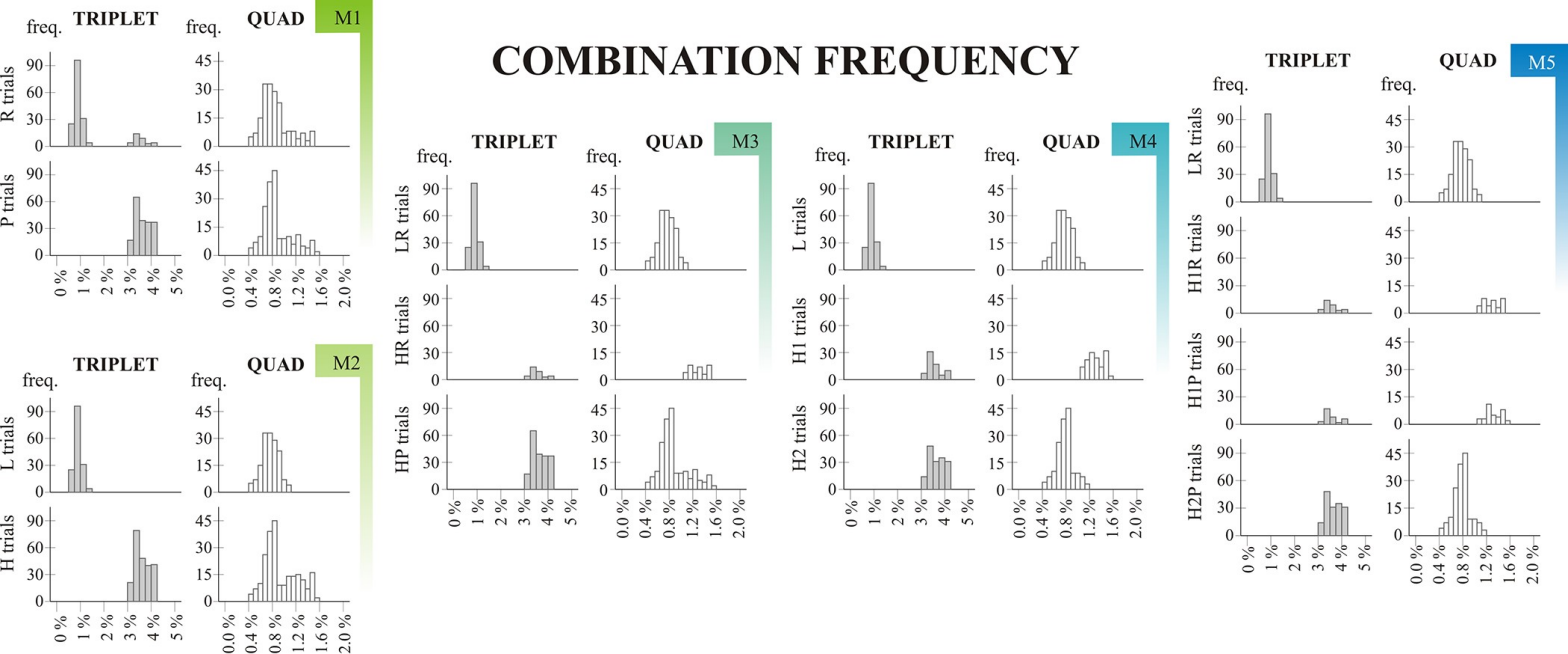
Combination frequency. M1 –Model 1; M2 –Model 2; M3 –Model 3; M4 –Model 4; M5 –Model 5. Combination frequency histograms are based on the ninth epoch (final ~400 trials) of a randomly chosen subject (subject number 111). The X axis shows the combination frequencies that occured in the given epoch of the ASRT task; the Y axis represent the frequency with which these occured. Two (triplet level) or three (quad level) preceding trials were taken into consideration when calculating joint probabilities (represented in different columns). Different rows represent different statistical categories within Models.

As described earlier, some combinations of consecutive trials are more frequent than others–and the longer combinations we assess, the more clusters we find, see Fig 3 for illustration. On this figure, histograms of combination frequencies are shown for each Model’s every category. It can be seen that from Model 1 to Model 5 the distributions are getting „narrower” (indicating a better categorization based on statistical properties).

Since learning scores are based on contrasting different categories within models, it is crucial that those categories should differ in combination frequencies that explicitly try to capture this aspect of learning (e.g. the H vs. L categories in Model 2; the LR vs. HR category of Model 3, the L vs. H1 categories of Model 4, and the LR vs. H1R categories of Model 5 when assessing triplet level learning; and the H1 vs. H2 categories of Model 4; and H1P vs. H2P categories of Model 5 when assessing quad learning). Some contrasts admittedly assess mixed effects, such as the HP vs. HR contrast of Model 3 and the Maximized Learning scores of Model 3–5.

As it can be seen on Fig 3 (and read from Table B in S1 File), these criteria are mostly met. However, for a small subset of participants, triplet level frequencies also differed between the HR vs. HP categories of Model 3 (~3% of participants); between the H1 and H2 categories of Model 4 (~ 4–8% of participants); between H1R and H1P categories of Model 5 (~2–4% of participants) and between H1P and H2P categories of Model 5 (~5–7% of participants). On a positive note, many of the previously discussed (predicted) effects were also confirmed; e.g. that Model 2 is better in capturing the triplet level statistical properties of the sequence than Model 1 (since effect sizes are higher for the former); and that the H1 vs. H2 distinction of Model 4 (and the H1P vs. H2P distinction of Model 5) also leads to higher quad level differences than the distinction HR vs. HP in Model 3 (for these statistics, see Table B in S1 File).

#### Trial probabilities (conditional probabilities)

Here the same conditions apply as described in the *Combination Frequencies* subsection since category boundaries are the same for joint frequencies and conditional probabilities (even though the direction of differences are not always the same). E.g. based on joint probabilities it could be expected that participants perform better on H1 than on H2 trials (since H1 combinations are more frequent than H2 combinations); based on conditional probabilities, however, better performance could be expected on H2 trials (since conditional probabilities are higher than for H1 trials).

**Fig 4:**
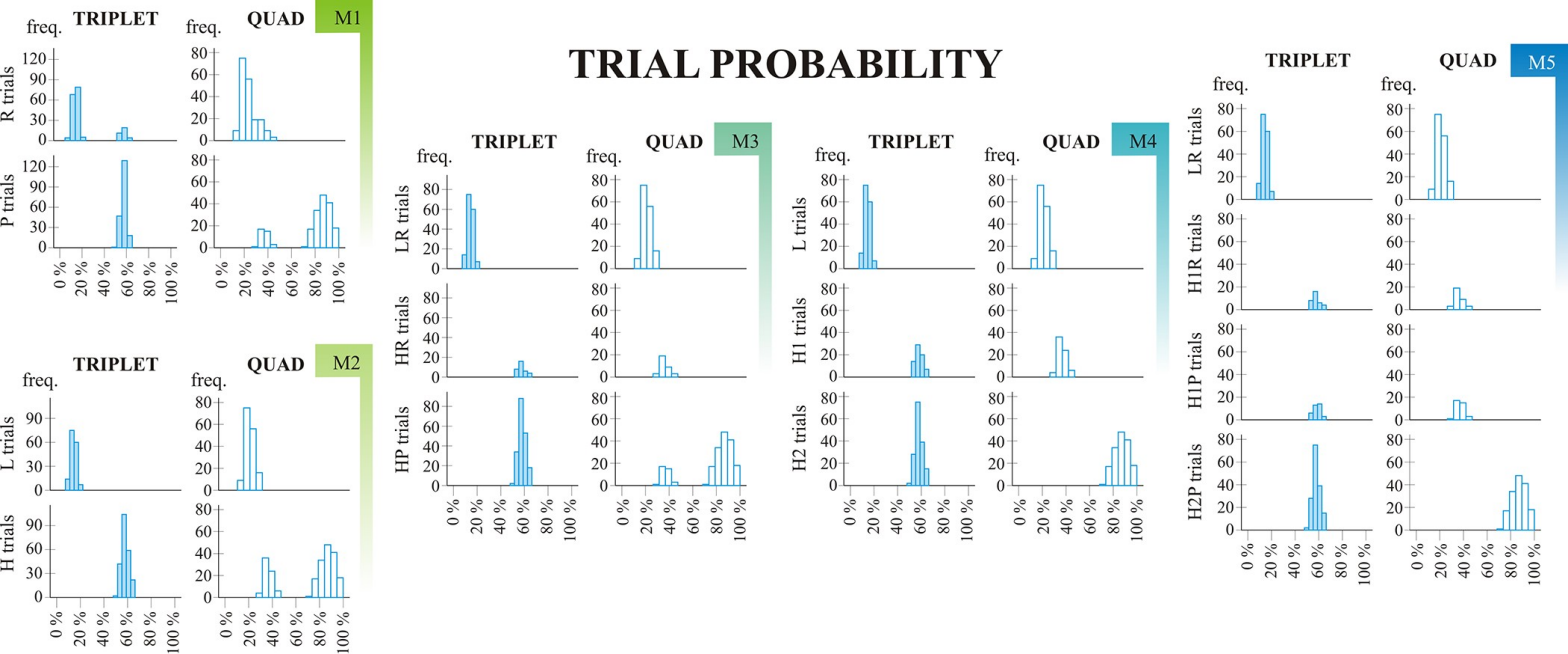
Trial probability. M1 –Model 1; M2 –Model 2; M3 –Model 3; M4 –Model 4; M5 –Model 5. Trial probability histograms are based on the ninth epoch (final ~400 trials) of a randomly chosen subject (subject number 111). The X axis shows trial probabilities that occured in the given epoch of the ASRT task; the Y axis represent the frequency with which these occured. Two (triplet level) or three (quad level) preceding trials were taken into consideration when calculating joint probabilities (represented in different columns). Different rows represent different statistical categories within Models.

The results are illustrated in Fig 4 and the corresponding statistics can be found in Table C in S1 File; these are all very similar to those discussed earlier at *Combination Frequencies*. As a plus, it was shown that quad level statistical information has a higher impact on Model 1 and Model 2 learning scores when assessing trial probabilities then when assessing combination frequencies (in line with the theoretical predictions; see Table B vs. Table C in S1 File).

#### Abstract structure of the combinations

**Fig 5:**
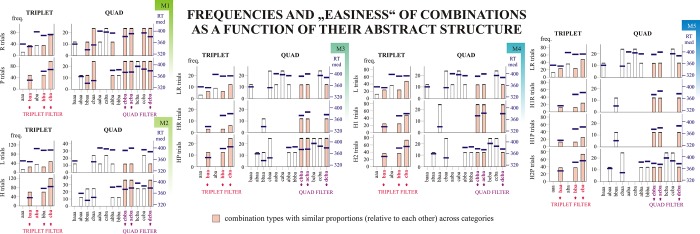
Abstract structure of the combinations. M1 –Model 1; M2 –Model 2; M3 –Model 3; M4 –Model 4; M5 –Model 5. The abstract structure of the combinations were defined the following way: the final trial of a combination was always denoted as *a*; the preceding trial as either *a* (if it was the same as the final trial) *or b* (in all other cases). If the N-2^th^ trial was identical to the N^th^ or N-1^th^ trial, the same notation was used as before (e.g. *a* or *b*), in all other cases a new notation was introduced (eg. *c*), etc. Bars indicate the mean number of category members in an epoch (~400 trials) calculated for each epoch of each participant. The black boxes at the top of the bars indicate the 95% confidence intervals of these means. The relative proportion of categories colored rose is identical in the Model’s subcategories. Dark blue boxes indicate the 95% confidence intervals of means of median RT values corresponding to the different categories (again, computed separately for each epoch of each participant). Red and purple arrows point to categories that are analyzed with Triplet Filter and Quad Filter, respectively.

Ideally, all of the categories (within models) should consist of similar combination types with regard to the combinations’ abstract structure, since pre-existing biases are not something we aim to measure in this task. The nevertheless existing differences can be reduced by applying filters (the usual Triplet Filter or the now-proposed, stricter, Quad Filter), see Fig 5. However, as can be read from Table D in S1 File, some differences remain. When the triplet filter is applied, and only three consecutive trials are considered, the most affected learning scores are those got by contrasting the P vs. R categories in Model 1 (affecting the scores of ~11% of participants), the HR vs. HP categories of Model 3 (~11% of participants); and the H1R vs. H1P categories of Model 5 (~15% of participants). By applying the stricter quad filter, the percentage of affected participants was reduced to 2%, 6%, and 5%, respectively. When considering four consecutive trials (i.e. quads), almost all of the learning scores are affected with Triplet Filtering (100% of participants showing such differences between the contrasted categories, with the exception of H1R vs. H1P learning in Model 5, which was only affected in 26% of participants). Evidently, by applying quad filtering, these numbers are greatly reduced. The most affected learning scores are the HR vs. HP learning in Model 3, and the H1R vs. H1P learning of Model 5 (affecting 14% and 19% of participants, respectively).

The fact that around 20% of participants show differences in combinations’ distribution (based on their abstract structure) for these Pattern Learning scores is important, and it should be taken as a warning to interpret these learning scores with caution.

In sum, only some of the learning scores fare well in measuring purely that factor that they aim to. The best model in this aspect is Model 5 which results in 4 (relatively) pure measures given that all factors contribute to learning. If, however, some of the information types are not picked up by participants, then less complex models could fare just as well as Model 5. For example, if pattern learning does not occur in the task (meaning that people do not differentiate pattern and random trials in addition to differences in statistical properties), then Model 4 should fare as good as Model 5. The question of which model results in highest explanatory power is assessed in the following section.

### Comparison of the models’ goodness of fit

As described in the previous section, Models 1–5 distinguish between an increasing number of categories (based on Trial Type and/or Statistical Information), and the main question is whether it’s worth to use the more elaborate models or there is no difference in how well they capture the essense of learning on the task. E.g. differentiating betweeen H1 and H2 trials (in Model 4 and Model 5) only makes sense if participants (or people, in general) are able to differentiate betweeen these categories when performing the task, which, on the other hand, should be reflected in differences in mean reaction times and/or accuracy. A way of assessing the goodness of fit of a model is by computing Adjusted R^2^ values (for reaction times data) and by computing Cramer’s Vs (for error data), and this is what we did for each model. If introducing or changing sub-categories explains the variability of data to a higher degree (i.e. there *is* a difference between H1 and H2 trials), the fit of the model will be higher, thus the goodness of models can be directly compared.

In this section the focus is on the goodness of models and not the effect of filtering; nevertheless we calculated the aforementioned effect sizes separately for each filtering method (no filter, triplet filter, quad filter), mainly to examine whether the change in effect sizes shows a similar pattern irrespective of the filtering being used.

#### Reaction times data

Each block consisted of 85 trials, five warm-up (random) trials and 80 ASRT trials (the alternation of pattern and random trials). Warm-up trials were not analysed, neither were trials 6–8 in a block (since it is only from trial 9 that the first full ASRT-quad is reached). This way 9.4% of trials were excluded. Out of the remaining trials, additional 18 percent was excluded due to errorneous responses on any of the quads trials (in other words, only those reaction time data points were analysed which corresponded to correct answers preceeded by another three correct answers in a row). Reaction times higher than 1000ms or lower than 150ms were also excluded from analysis (0.1 percent of the remaining data). Additionally, reaction times having a Z score higher than 2 or lower than -2 were removed from each epoch from each statistical category of the most sophisticated Model (i.e. Model 5, LR, H1R, H1P and H2P) for each participant to minimize the effect of outliers. This way 4.5% of the remaining data was removed when using no filter; 16% when using the triplet filter and 64.6% when using the quad filter (the high percentage of excluded trials using the triplet filter and quad filter results from the filters themselves, not from so many Z-scores having a high absolute value). At the end, an average of 2710 trials were analysed per participant when using no filter; an average of 2384 trials when using triplet filter and an average of 1006 trials when using the quad filter.

We computed individual adjusted R^2^-s for each epoch of each participant as a way of assessing the goodness of fit of each Model; since there were nine epochs, this resulted in nine values per participant. These were than averaged to yield a single value for everyone. The effect of different filtering methods was also taken into account by computing these effect sizes for each filtering type separately (No Filter, Triplet Filter and Quad Filter). The goodness of fits were then compared by a FILTER TYPE (3 levels: No Filter, Triplet Filter, Quad Filter) x MODEL (5 levels: Model 1—Model 5) Repeated Measures ANOVA. Sphericity was assessed with Mauchly’s Test, and if this precondition was not met, degrees of freedom were adjusted with the Greenhouse-Geisser method. Bonferroni-corrected post hoc tests were performed whenever the omnibus ANOVA showed significant main effects or interactions. Partial eta squared effect sizes are reported in line with significant main effects or interactions in the ANOVA.

**Fig 6:**
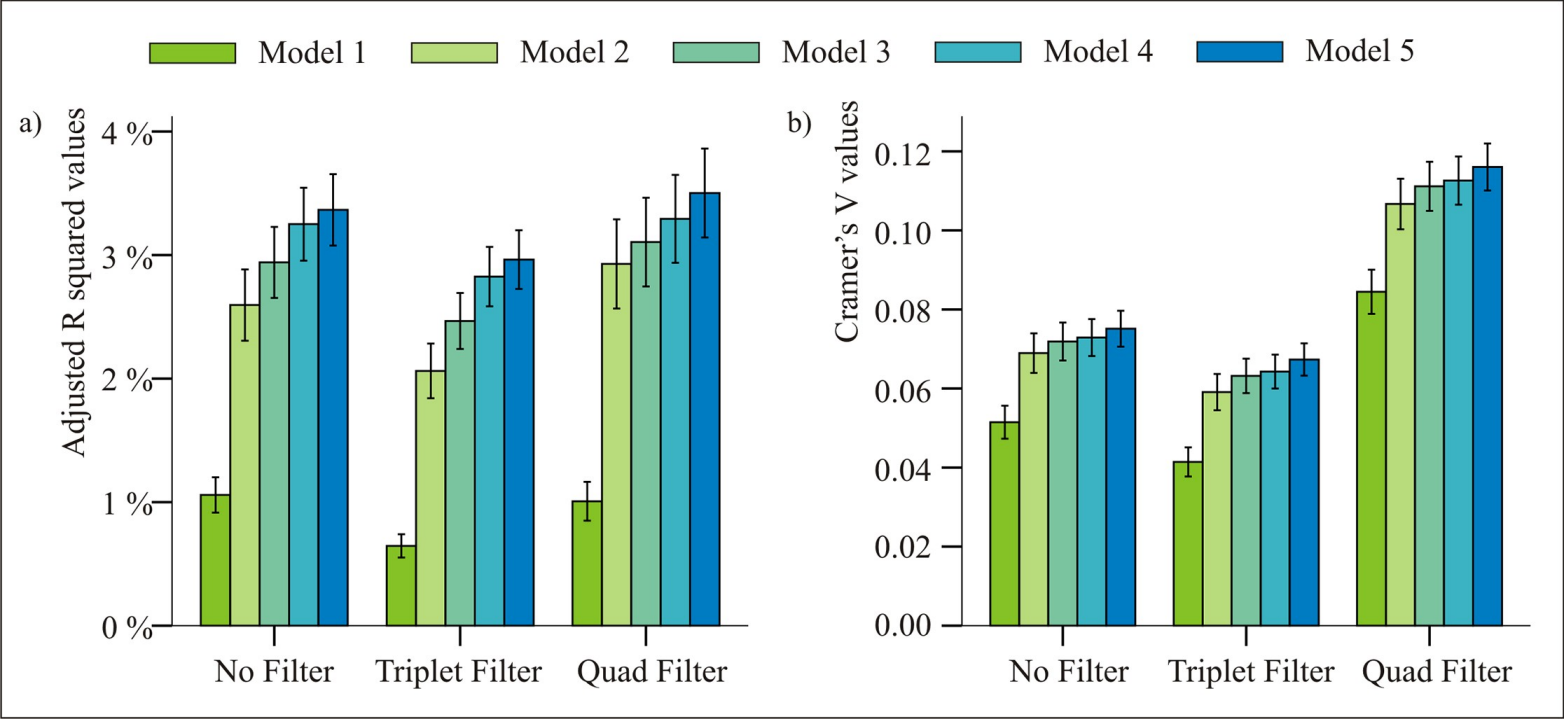
Goodness of fit of the different models within each filtering method. a) Individual Adjusted R2 values based on reaction times. Each Model differed from all the other Models within each filtering method (all p < 0.012). b) Individual Cramer’s V values based on error data. Each Model differed from all the others within each filtering method, except for the differences Model3 vs. Model4 (no filter p = 0.166, triplet filter p = 0.359, quad filter p = 0.261). Error bars are 95% confidence intervals.

The main effect of FILTER TYPE was significant, F(1.553, 278.066) = 25.562, MSE < 0.001, p < 0.001, *η*_*p*_^*2*^ = 0.125, indicating that, on average, the goodness of fits differed as a function of the filter used. These differences were better captured with a quadratic model than with a linear one (p < 0.001 vs. p = 0.190 and *η*_*p*_^*2*^ = 0.271 vs. *η*_*p*_^*2*^ = 0.010 respectively). Bonferroni corrected post hoc tests revealed that means of adjusted R squared values were highest with the Quad Filter and lowest with the Triplet Filter, all contrasts being significant (p < 0.001) except for the contrast No Filter vs. Quad Filter (p = 0.569). The main effect of MODEL was also significant, F(1.384, 247.759) = 408.371, MSE < 0.001, p < 0.001, *η*_*p*_^*2*^ = 0.695, indicating that model goodness of fits differed as a function of the Model used in the analysis. These differences was best explained with a linear model (as compared to quadratic, cubic or higher order models; *η*_*p*_^*2*^ = 0.778 for the linear model, and *η*_*p*_^*2*^ < 0.555 for the other models), as values grew monotonicaly from Model 1 to Model 5. Bonferroni corrected post hoc tests revealed that all paired comparisons were significant (all p < 0.001). Finally, the interaction of FILTER TYPE x MODEL was also significant, F(2.492, 446.058) = 11.122, MSE < 0.001, p < 0.001, *η*_*p*_^*2*^ = 0.058, indicating that the monotonic growth of adjusted R squared values as a funtion of MODEL were not equivalent with the three filtering methods used. Bonferroni corrected post hoc tests revealed that each Model differed from all the others within each filtering method (all p < 0.012). The effect of the differing filters was also quite consistent with each Model, showing that both the No Filter condition and the Quad filter condition yielded higher fits than the Triplet Filter condition (all p < 0.001), the Quad Filter and No Filter condition not differing from each other in 4 out of 5 cases (all p > 0.437, except for Model 2 where p = 0.006). The results are shown on Fig 6A). A more fine grained, epoch-by-epoch analysis of adjusted R^2^ values is shown on Figure A in S1 File.

#### Errors

Warm-up trials were not analysed, neither were trials 6–8 in a block (since it is only from trial 9 that the first full ASRT-quad is reached). This way 9.4% of trials were excluded. Additionaly, only those data points were included that were preceeded by at least three correct responses in a row (this way it could be ensured that the sequence of buttonpresses corresponded to the intended combinations before a critical trial); this resulted in the removal of additional 13.9% of the remaining trials. When no filtering was applyied, an average of 2981 trials were analysed per participant; triplet filtering resulted in an average of 2610 trials, while quad filtering in an average of 1113 trials per parcitipant. The goodness of fit of the different models were than calculated in the form of Cramer’s V values (data from the nine epochs were collapsed into a single category due to the small number of errors) separately for each filtering method. To compare the obtained Cramer V values, we run a FILTER TYPE (3 levels: No Filter, Triplet Filter, Quad Filter) x MODEL (5 levels: Model 1—Model 5) Repeated Measures ANOVA. Sphericity was assessed with Mauchly’s Test, and if this precondition was not met, degrees of freedom were adjusted with the Greenhouse-Geisser method. Bonferroni-corrected post hoc tests were performed whenever the omnibus ANOVA showed significant main effects or interactions. Partial eta squared effect sizes are reported in line with significant main effects or interactions in the ANOVA.

The main effect of FILTER TYPE was significant, F(1.472, 263.495) = 489.885, MSE = 0.002, p < 0.001, *η*_*p*_^*2*^ = 0.732, indicating that, on average, the goodness of fits differed as a function of the filter used. These differences were better captured with a quadratic model than with a linear one (both p < 0.001, but the effect size for the quadratic model is *η*_*p*_^*2*^ = 0.828, while it is *η*_*p*_^*2*^ = 0.676 for the linear model). Bonferroni corrected post hoc tests revealed that means of Cramer V values were highest with the Quad Filter and lowest with the Triplet Filter, all contrasts being significant (p < 0.001). The main effect of MODEL was also significant, F(1.598, 286.124) = 281.264, MSE = 0.001, p < 0.001, *η*_*p*_^*2*^ = 0.611, indicating that model goodness of fits differed as a function of the Model used in the analysis. These differences were best explained with a linear model (as compared to quadratic, cubic or higher order models, *η*_*p*_^*2*^ = 0.688 for the linear model, and 0.503, 0.470 and 0.047 for the higher order models, respecitvely); values grew monotonicaly from Model 1 to Model 5. Bonferroni corrected post hoc tests revealed that all paired comparisons were significant (all p < 0.001) except for the difference between Model3 and Model4 (p = 0.231). Finally, the interaction of FILTER TYPE x MODEL was also significant, F(1.747, 312.721) = 40.517, MSE < 0.001, p < 0.001, *η*_*p*_^*2*^ = 0.185, indicating that the monotonic growing of adjusted R squared values as a funtion of MODEL were not equivalent with the three filtering methods used. Bonferroni corrected post hoc tests revealed that each Model differed from all the others within each filtering method, except for the differences Model3 vs. Model4 (no filter p = 0.166, triplet filter p = 0.359, quad filter p = 0.261). The effect of the differing filters was also quite consistent with each Model, showing all filtering methods differed from the rest (all p < 0.001). The results are shown on Fig 6B).

### Comparison of the filters

#### Mean reaction times and error percentages belonging to the models’ categories

To get a more sophisticated picture, we calculated the mean reaction times and error percentages broken down by the categories specified by the Models separately for each filtering method. This was done for each of the nine epochs for each participant, and then these nine values were averaged to yield a single value for each cell for each participant. We summarized the means of these mean reaction times and mean error percentages, standard deviations (SD) of these means and the coefficients of variations of these means (CV = SD/mean in %) (Table E in S1 File).

Does filtering alter mean reaction times corresponding to the categories specified by a certain model? To answer this question, we ran Repeated Measures ANOVAs with FILTERING (no filter, triplet filter, quad filter) as an independent variable (and categories’ means as dependent variables). Our results showed that all category means differed as a function of FILTERING (all *p* < 0.001, all *η*_*p*_^*2*^ > 0.255) except for the H2 and H2P categories (in Model4 and Model5, respectively; *p* = 0.998, *η*_*p*_^*2*^ < 0.001). In cases of significant omnibus ANOVAs, Bonferroni corrected post hoc tests were run. Triplet filtering, in contrast to no filtering, altered the mean reaction times in the *random* (R) category in Model 1 (*p* < 0.001), and low-frequency triplets’ reaction times in Model 2–5 on a trend level (*p* = 0.095). In all these cases, means got lower. Quad filtering, on the other hand, increased means in all of the categories in each Model (both relative to no filtering and relative to triplet filtering, all *p* < 0.001), which indicates that, predominantly, „easy” combinations had been eliminated with this filter (see also Fig 5 for a similar conclusion).

A very similar pattern emerged with Repeated Measures ANOVAs performed on the mean percentages of errors: a significant main effect of FILTERING was observed for each category of each Model (all p < 0.001, all *η*_*p*_^*2*^ > 0.113) except for the H2 and H2P categories of Model4 and Model5, respectively (p = 0.412, *η*_*p*_^*2*^ = 0.005). In cases of significant omnibus ANOVAs, Bonferroni corrected post hoc tests were run. Triplet filtering, in contrast to no filtering, altered (lowered) the mean error percentages in the *random* (R) category in Model 1 and low-frequency triplets’ reaction times in Model 2–5 (all *p* < 0.001). Quad filtering, on the other hand, increased mean percentages of errors (both in contrast to no filtering and triplet filtering), and this increase was significant in all but one of the cases (all p < 0.001; except for the R category of Model1 where p > 0.999).

#### Learning effects

Solely the fact that mean reaction times and error percentages are subject to change when not all data is included is not surprising, and, in itself, not very meaningful. The real question is whether *learning effects* are subject to change when we apply different filters (e.g. whether category means change in parallel or some are affected more than others, or in other directions than others, resulting in changed learning scores as well). To answer this question, we calculated all the possible learning effects in the form of Cohen’s d-s (RT data) and Cramer’s V-s (error data) for each Model and each filtering method individually. In the case of reaction times, these effect sizes were calculated separately for the nine epochs and then averaged for each participant; in the case of errors, the data from the nine epochs were pooled for each participant (due to very low numbers of errors), and thus only one Cramer’s V effect size was calculated per cell. Table F (in S1 File) summarizes the means of the individual effect sizes, the SD of these means and the CV of these means. Positive values indicate that the difference between categories showed the expected pattern, while negative values indicate the opposite (e.g. the easier/more predictable trials being responded to slower or less accurately), usually an unexpected result. As an exception, the contrasts H1 vs. H2 in Model 4 and H1P vs. H2P in Model 5 might result in negative values if joint probabilty learning is higher/more dominant than conditional probability learning, thus they couldn’t automatically be considered *false negatives*.

#### Specific learning effects based on reaction times

To assess whether the filtering method had an effect on individual effect sizes, we first run Repeated Measures ANOVA-s on the Cohen’s d values obtained for all the possible learning measures of the five Models with FILTER (no filter, triplet filter, quad filter) as an independent variable. Filter had an effect in all cases (all p < 0.001, all *η*_*p*_^*2*^ > 0.164), except for the *pattern learning* measure of Model5 (H1P vs. H1R), which remained unchanged (p = 0.626, *η*_*p*_^*2*^ = 0.003). In cases of significant omnibus ANOVAs, Bonferroni corrected post hoc tests were run. Triplet filtering (in contrast to no filtering) left some of the learning measures unaffected (those that are based solely on high-frequency triplets; i.e. HR vs. HP in Model 3; H1 vs. H2 in Model4; H1P vs. H2P and H1R vs. H1P in Model5). In all the remaining cases individual effect sizes decreased as a result of triplet filtering (all *p* < 0.048). Quad filtering, on the other hand, resulted in mixed effects. It increased effect sizes obtained in the simple models Model1 and Model2 (P vs. R; H vs. L–both of which could be considered quite mixed effects), and in the more elaborated Models (3–5) it increased those effects that depicted higher order statistical learning measures (HR vs. HP in Model3, H1 vs. H2 in Model4; and H1P vs. H2P in Model5; all *p* < 0.001). It is worth noting that some of these values not only increased but reversed their direction when applying the quad filter, leading to qualitatively different conclusions about learning. At the same time when higher order statistical learning effects increased, effects that depict the relatively pure measure of triplet level statistical learning (LR vs. HR in Model3; L vs. H1 in Model4; and LR vs. H1R in Model5) decreased in contrast to other filtering options when applying the quad filter (all *p* < 0.001). Learning measures depicting *maximum learning* (HP vs. LR in Model3, H2 vs. L in Model4 and H2P vs. LR in Model5) showed a significant increase (all p < 0.001), but its worth remembering that this is admittedly a mixed effect showing the summarized changes in different measures of each Model.

#### Specific learning effects based on errors

Error data showed a similar (although not identical) pattern. In this case, the dependent variables were the Cramer’s V values, the independent variable was again the way of filtering data (FILTER: no filter, triplet filter, quad filter). The main effect of FILTER was significant in most of the cases (all p < 0.006, all *η*_*p*_^*2*^ > 0.037) except for the *triplet learning effects* HR vs. LR in Model3 and its equivalent H1R vs. LR in Model5 (*p* = 0.584, *η*_*p*_^*2*^ = 0.002) and the *pattern learning* effect of Model5 (H1P vs. H1R, p = 0.335, *η*_*p*_^*2*^ = 0.006). In cases of significant omnibus ANOVAs, Bonferroni corrected post hoc tests were run. Triplet filtering, in contrast to no filtering, decreased effect sizes in all cases except for those that are by definition unaffected by triplet filtering (all *p* < 0.002). Quad filtering, on the other hand, had a differential effect on learning measures depending on what kind of learning they depicted. Again, learning scores associated with higher order statistical learning (HR vs. HP in Model3; H1 vs. H2 in Model4; H1P vs. H2P in Model5) showed an increase when the quad filter was applied (all p < 0.001)–and, again, these effects reversed their directions from being on average negative to being on average positive. At the same time, the learning measure depicting pure triplet level learning (L vs H1 in Model4) decreased (all p < 0.004). Effect sizes of the relatively simple models Model1 and Model2 (showing quite mixed effects) also decreased when the quad filter was applied, but this decrease was only significant relative to the *no filter* condition (both p < 0.002) but not relative to the triplet filtering condition (both p > 0.999). *Maximum Learning* effects of Model3, Model4, and Model5 also showed somewhat mixed effects. When the quad filter was applied, there was an increase in effect sizes relative to triplet filtering in the case of Model4 and Model5 (both p < 0.001). None of the remaining contrasts approached significance (all *p* > 0.177). The mixed effects of *Maximized Learning* measures are not of a surprise since these reflect the sum of the positive and negative changes of individual learning measures of each Model.

#### Variability

It is crucial to be able to detect individual differences (i.e. between-subjects variability) with any task. One way of doing so is to assess the variability of individual learning scores (e.g. standard deviation, SD). If variability decreases with a stricter filtering, it might indicate that some of the differences seen in previous studies are attributable to differences in pre-existing biases to certain movement combinations. If, however, variability is increased when using a stricter filtering, it might mean that some of the variability in implicit learning capabilities were previously masked due to the systematic noise attributable to pre-existing tendencies. Importantly, this kind of variability may also increase as a result of increased noise (i.e. less precise estimates) on the individual level when a stricter filtering comes with a smaller number of trials being analysed.

#### How does filtering affect the variability of the learning scores?

To test the homogeneity of variances, Levene-test was applied on individual learning scores with FILTERING (No Filter, Triplet Filter, Quad Filter) as an independent variable (for this particular analysis treated as a between-subjects variable). According to the test, filtering had a significant effect on variances in most of the cases (p < 0.032); the exceptions were Higher Order Learning score of Model 3; F(2, 537) = 1.683, p = 0.187; and the Quad Learning score in Model 4; F(2, 537) = 1.977, p = 0.140. To unpack the observed differences, we also computed the Levene test in pairs (No Filtering vs. Triplet Filtering; No Filtering vs. Quad Filtering and Triplet Filtering vs. Quad Filtering). The results are shown in Table 1.

**Table 1:** Between-subjects variability of individual learning scores as a function of filtering.

		Using the Triplet Filter (as opposed to No Filter)			Using the Quad Filter (as opposed to No Filter)			Using the Quad Filter (as opposed to Triplet Filter)		
		Variance change ↓ decrease ↑ increase	Levene test F value	Levene test p value	Variance change ↓ decrease ↑ increase	Levene test F value	Levene test p value	Variance change ↓ decrease ↑ increase	Levene test F value	Levene test p value
M1	Trial Type Effect	↓	**11.019**	0.001*	↓	1.064	0.303	**↑**	**4.913**	0.027*
M2	Sequence Specific L.	↓	**4.892**	0.028*	**↑**	**9.386**	0.002*	**↑**	**26.676**	<0.001*
M3	Pure Statistical Learning	↓	0.919	0.338	**↑**	**7.412**	0.007*	**↑**	**13.095**	<0.001*
	Higher Order Seq. Learn.	=	0.000	> 0.999	↑	2.451	0.118	↑	2.451	0.118
	Maximized Learning	**↓**	**5.433**	0.020*	**↑**	**7.258**	0.007*	**↑**	**23.779**	<0.001*
M4	Triplet Learn. (+ Pattern L.)	↓	1.131	0.288	**↑**	**7.587**	0.006*	**↑**	**14.805**	<0.001*
	Quad Learn (+ Pattern L.)	=	0.000	> 0.999	**↑**	**4.872**	0.028*	**↑**	**4.872**	0.028*
	Maximized Learning	**↓**	**6.465**	0.011*	**↑**	**7.443**	0.007*	**↑**	**24.574**	<0.001*
M5	Triplet Learning	↓	0.919	0.338	**↑**	**7.412**	0.007*	**↑**	**13.095**	<0.001*
	Pattern Learning	=	0.000	> 0.999	**↑**	**9.822**	0.002*	**↑**	**9.822**	0.002*
	Quad Learning	=	0.000	> 0.999	**↑**	***2*.*734***	***0*.*099***^***+***^	**↑**	***2*.*734***	***0*.*099***^***+***^
	Maximized Learning	**↓**	**6.465**	0.011*	**↑**	**7.443**	0.007*	**↑**	**24.574**	<0.001*

M1-M5: Model 1 –Model 5

* significant difference, p < .05

+ tendency towars significance, p < .10

#### Is higher variability caused by less precise estimates?

Standard deviations (SD) of the learning scores and the coefficients of variations (CV) are shown in Table F in S1 File. The fact that CVs show a similar pattern to SDs indicates that higher standard deviations are not only a straightforward consequence of higher means. Moreover, in the case of triplet learning measures, a typical result is that decreased means are associated with increased standard deviations (e.g. Model 2 L-H, Model 3 LR-HR, Model 4 L-H1, Model 5 LR-H1R). This pattern of results was not only consistent across reaction times and error data (see Table F in S1 File), but also across the six ASRT sequences when assessed separately (SD and CV in Tables H and I in S1 File and Tables K and L in S1 File; while Table G and Table J in S1 File shows the means of individual effect sized broken down by the six possible ASRT sequences. Note that CV values could be inflated in cases when means approach zero).

Why did these differences arise? In an optimistic scenario, they are the result of quad filtering making it possible for us to detect previously undetectable (masked) individual differences in learning capabilities. In a pessimistic case, however, higher variability stems from other sources. For example, it may be a consequence of noisier estimates on the individual level since the number of analyzed trials is smallest with quad filtering. In order to check up on this possibility, we calculated the within-subject standard deviations (SD) and coefficients of variations (CV) of reaction times for each epoch and each statistical category for each participant. We averaged the values obtained for the nine epochs in order to get a single value for each category for each participant (see Table M in S1 File). Note that we could not compute standard deviations (and CVs) for error data within individuals since accuracy is a binary data type (a particular press is either correct or incorrect).

We ran Repeated Measures ANOVAs on these SD values with FILTER (no filter, triplet filtering, quad filtering) as a within-subject factor. The main effect was significant every time (all p < 0.001, all *η*_*p*_^*2*^ > 0.124). To disentangle these omnibus effects, Bonferroni corrected post hoc tests were run. Triplet filtering (contrasted with no filtering) left some of the SD-s unaffected (since, by definition, triplet filtering does not affect high-frequency triplets). In all the remaining categories, SDs decreased as a result of triplet filtering (all p < 0.012). Quad filtering resulted in further decreases in all categories (even those unaffected by triplet filtering), all p < 0.001. This effect was quite consistent, as participants have shown the previosly described pattern in (on average) 5–6 epochs out of 9 (see Table 2).

**Table 2:** Within-subject variability of the estimates (that the learning scores are based on) as a function of filtering.

Model	Category	Mean Number of Epochs in which the SD decreased with the use of a particular Filter for a given individual *(SD of the Mean)*			Mean Number of Epochs in which the CV decreased with the use of a particular Filter for a given individual *(SD of the Mean)*		
		TF compared to NF	QF compared to NF	QF compared to TF	TF compared to NF	QF compared to NF	QF compared to TF
M1	R	**4.66** *(2*.*03)*	**5.88** *(1*.*97)*	**5.61** *(2*.*03)*	**4.23** *(1*.*97)*	**6.27** *(2*.*08)*	**6.17** *(2*.*22)*
	P	*No* *Diff*.	**5.69** *(1*.*97)*	**5.69** *(1*.*97)*	*No* *Diff*.	**5.82** *(2*.*11)*	**5.82** *(2*.*11)*
M2	L	**4.50** *(1*.*97)*	**5.12** *(2*.*09)*	**5.03** *(2*.*11)*	**4.53** *(1*.*92)*	**5.74** *(2*.*18)*	**5.63** *(2*.*16)*
	H	*No* *Diff*.	**5.83** *(2*.*02)*	**5.83** *(2*.*02)*	*No* *Diff*.	**6.18** *(2*.*10)*	**6.18** *(2*.*10)*
M3	LR	**4.50** *(1*.*97)*	**5.12** *(2*.*09)*	**5.03** *(2*.*11)*	**4.53** *(1*.*92)*	**5.74** *(2*.*18)*	**5.63** *(2*.*16)*
	HR	*No* *Diff*.	**5.53** *(2*.*16)*	**5.53** *(2*.*16)*	*No* *Diff*.	**6.47** *(2*.*14)*	**6.47** *(2*.*14)*
	HP	*No* *Diff*.	**5.69** *(1*.*97)*	**5.69** *(1*.*97)*	*No* *Diff*.	**5.82** *(2*.*11)*	**5.82** *(2*.*11)*
M4	L	**4.50** *(1*.*97)*	**5.12** *(2*.*09)*	**5.03** *(2*.*11)*	**4.53** *(1*.*92)*	**5.74** *(2*.*18)*	**5.63** *(2*.*16)*
	H1	*No* *Diff*.	**5.88** *(2*.*37)*	**5.88** *(2*.*37)*	*No* *Diff*.	**6.72** *(2*.*24)*	**6.72** *(2*.*24)*
	H2	*No* *Diff*.	**5.38** *(1*.*83)*	**5.38** *(1*.*83)*	*No* *Diff*.	**5.32** *(2*.*03)*	**5.32** *(2*.*03)*
M5	LR	**4.50** *(1*.*97)*	**5.12** *(2*.*09)*	**5.03** *(2*.*11)*	**4.53** (1.92)	**5.74** *(2*.*18)*	**5.63** *(2*.*16)*
	H1R	*No* *Diff*.	**5.53** *(2*.*16)*	**5.53** *(2*.*16)*	*No* *Diff*.	**6.47** *(2*.*14)*	**6.47** *(2*.*14)*
	H1P	*No* *Diff*.	**5.54** *(2*.*20)*	**5.54** *(2*.*20)*	*No* *Diff*.	**6.37** *(2*.*13)*	**6.37** *(2*.*13)*
	H2P	*No* *Diff*.	**5.38** *(1*.*83)*	**5.38** *(1*.*83)*	*No* *Diff*.	**5.32** *(2*.*03)*	**5.32** *(2*.*03)*

M1-M5: Model 1 –Model 5

NF: No Filter, TF: Triplet Filter, QF: Quad Filter

No Diff: Filtering did not affect the category, thus no difference could be observed

The decrease of standard deviations should not be attributed to decreased means exclusively since some of the means actually increased with the use of filters (see Table E in S1 File). To corroborate this thought, we conducted the previously described ANOVAs on CVs, too. The main effect of FILTERING was, again, significant in all cases, all p < 0.001, all *η*_*p*_^*2*^ > 0.189. Bonferroni corrected post hoc tests showed that triplet filtering has left these values largely unaffected (except for the L and LR categories of Model2, Model3, Model4, and Model5; a slight decrease on a trend level, p = 0.090). Quad filtering, on the other hand, decreased CV-s in all cases (all p < 0.001), indicating that, in this case, standard deviations decreased more than the means. This result clearly indicates that higher individual differences are not caused by noisier estimates of mean reaction times on the individual level. Importantly, this pattern was not specific to a subset of the ASRT sequences, as it was observed in all of them (see Tables N and O in S1 File).

### Does higher variability go in hand with lower reliability?

Reliability assesses whether the outcome of a test would be similar when repeated. Unsystematic noise decreases reliability, while systematic patterns in variation increase it. Thus, the higher the systematic variation in our data (relative to unsystematic noise), the higher the reliability indices will be. The problem here is that pre-existing biases–that we aim to reduce with more strict filtering methods–may actually introduce systematic variability rather than unsystematic noise. That being said, it is entirely possible that by reducing the variability that is attributable to pre-existing biases, reliability indices drop; and this is exactly what we have found.

We calculated split-half reliability of all of the measures by randomly assigning each keypress to one of two categories; the individual effect sizes were then computed for both sets, and the correlation of the two values was computed. In the case of reaction times, individual Cohen’s d-s were calculated for each Session (Epoch 1–3, Epoch 4–6 and Epoch 7–9) rather than for each Epoch separately in order to compensate for the low number of trials per epoch when the data is split in two, and the three values were then averaged to yield a single effect size for both sets. In the case of accuracy, a single Cramer’s V was calculated for Epochs 1–9 for both sets (data from the nine epochs collapsed due to low overall error rates). Split-half reliabilities for reaction times are shown in Table 3.

**Table 3:** Split-half reliability of each of the possible learning scores (Models 1–5, all filtering types) based on reaction times.

		Reaction Times			Accuracy		
		No Filter	Triplet Filter	Quad Filter	No Filter	Triplet Filter	Quad Filter
M1	**R-P** *TrialType effect*	.770**	.545**	.365**	.514**	.354**	.267**
M2	**L-H** *Seq*. *Spec*. *L*.	.843**	.713**	.614**	.576**	.459**	.416**
M3	**LR-HR** *Pure Stat*. *L*.	.691**	.630**	.556**	.356**	.336**	.270**
	**HR-HP** *Higher Ord*. *L*.	.366**	.366**	.236**	.137	.137	.025
	**LR-HP** *Max*. *Learning*	.835**	.687**	.581**	.573**	.446**	.400**
M4	**L-H1** *Triplet L*.*+ P*. *L*.	.788**	.707**	.603**	.489**	.414**	.347**
	**H1-H2** *Quad L*.*+ P*. *L*.	.595**	.595**	.374**	.176*	.176*	.008
	**L-H2** *Max*.*Learning*	.828**	.680**	.538**	.535**	.423**	.337**
M5	**LR-H1R** *Triplet Learning*	.691**	.630**	.556**	.356**	.336**	.270**
	**H1R-H1P** *Pattern Learning*	.090	.090	.226**	.025	.025	.041
	**H1P-H2P** *Quad Learning*	.477**	.477**	.363**	.078	.078	.022
	**LR-H2P** *Max*. *Learning*	.828**	.680**	.538**	.535**	.423**	.337**

* p < .05

** p < .01

We assigned each trial one of two possible codes, and the resulting two sets were analysed separately (thus learning, fatigue, etc. affected each set similarly). In the case of reaction times, learning scores were computed for each Session (epochs 1–3, epochs 4–6 and epochs 7–9), and then averaged. In the case of accuracy, a single Cramer’s V was calculated for Epochs 1–9 for both sets (data from the nine epochs collapsed due to low overall error rates). The correlation between the two subsets is shown in the table (Pearson correlation coefficients).

As can be seen, reliability indices dropped substantially when using the quad filter. This may be attributable to the possibility that pre-existing biases are a form of a systematic artifact (rather than noise), as noted earlier. Conversely, it is also possible that the drop is attributable to increased levels of noise (since fewer trials are analyzed with stricter filtering). However, even these lower indices are not surprising (at least for reaction times data), as the reliability of implicit learning measures is often low [48], and difference scores might also have lower reliabilities than the components they are derived from, proportionally to the correlation between the original components [49]. As a comparison, Kaufman et al. used a probabilistic SRT task that is in many aspects similar to the ASRT used in our study, and the split-half reliability of the RT difference score was 0.33 [50]. It is worth emphasizing that reliability is a different concept from validity, i.e. whether we measure what we aim to measure. For methodological reasons, we argue that quad filtering makes the ASRT a less reliable but more valid task for assessing implicit learning.

### New insights—What is being learned ASRT task?

After arguing for the use of the newly proposed analysis methods (Model 5 and Quad Filtering), we would like to briefly review its results. The focus here is not whether results differ from those gained using the typical analysis (they do, see above), but purely descriptively: is there evidence for quad learning, triplet learning and pattern learning on the group level? What percentage of participants show learning on these measures?

In the case of reaction times, the first question was assessed with a Repeated Measures ANOVA, with Epochs (1–9) as an independent variable and individual Cohen’s d values (in each epoch) as the dependent variable. This way both the overall learning and its time course were assessed. In the case of error data, time course couldn’t be taken into account due to the low overall number of errors. Thus, in this case, a single Cramer’s V value was calculated for each participant (data of Epoch 1–9 collapsed into a single category), and these values were compared to zero using a one-sample t-test.

To assess the second question, we identified participants with individual Cohen’s d values and Cramer’s V values exceeding the limit of 0.2 and 0.05, respectively. We also quantified the percentage of participants whose effect sizes exceeded these limits but the difference was in the unexpected direction (presumably false negatives). If the ratio of false positives is similar to the ratio of false negatives, then the percentage of true positives could be gauged by subtracting the (false) negatives from the positives. In the case of reaction times, we also calculated the percentage of *reliable learners* by identifying participants who had shown at least a small learning (Cohen’s d > 0.2) in at least 5 (out of 9) epochs. Such a detailed analysis was not possible in the case of accuracy data due to the low overall error rates.

For error data, Individual effect sizes (Cramer’s V-s) were calculated for the collapsed data of the nine epochs because of the low number of errors (consequently, the time-course of learning could not be examined). Since these effect sizes represent some kind of an individual average, maximum values are expected to be lower than RT-related effect sizes. For this reason, we decided to use Cramer’s V > 0.05 as an indication of small learning (instead of the conventional Cramer’s V > 0.10). Our results are presented in Table 4.

**Table 4:** Average learning scores and the percentage of participants who learned particular types of information.

		Triplet Learn.	Quad Learn.	Pattern Learn.	Max Learn.
RT data	Overall learning (descriptive statistics, and the ANOVA’s intercept)	*M* = 0.350, *SEM* = 0.014; *F*(1,179) = 597.78, *MSE* = 0.331, *p* < 0.001, *η*_*p*_^*2*^ = 0.770	*M* = 0.056, *SEM* = 0.010; *F*(1,179) = 30.79, *MSE* = 0.163, *p* < 0.001, *η*_*p*_^*2*^ = 0.147	*M* = -0.028, *SEM* = 0.009; *F*(1, 179) = 10.72, *MSE* = 0.122, *p* = 0.001, *η*_*p*_^*2*^ = 0.057	*M* = 0.376, *SEM* = 0.014; *F*(1, 179) = 767.92, *MSE* = 0.298, *p* < 0.001, *η*_*p*_^*2*^ = 0.811
	ANOVA’s main effect of EPOCH	*F*(8, 1432) = 23.03, *MSE* = 0.108, *p* < 0.001, *η*_*p*_^*2*^ = 0.114	*F*(8, 1432) = 2.09, *MSE* = 0.104, *p* = 0.033, *η*_*p*_^*2*^ = 0.012	*F*(8, 1432) = 1.27, *MSE* = 0.097, *p* = 0.251, *η*_*p*_^*2*^ = 0.007	*F*(7, 1333) = 32.77, *MSE* = 0.097, *p* < 0.001, *η*_*p*_^*2*^ = 0.155
	% of positive learners (% of reliably positive learners)	76.77% (74.44%)	12.22% (13.33%)	5.00% (5.56%)	87.22% (83.89%)
	% of negative learners (% of reliably negative learners)	0.00% (0.00%)	4.44% (5.00%)	4.44% (10.56%)	0.00% (0.00%)
	% of true learners (% of reliably true learners)	76.77% (74.44%)	7.78% (8.33%)	0.56% (-5.00%)	87.22% (83.89%)
Accuracy data	Overall learning (descriptive statistics, and the results of the t-test)	*M* = 0.057, *SEM* = 0.004, *t*(179) = 13.766, *p* < 0.001, *Cohen’s d* = 1.026	*M* = 0.012, *SEM* = 0.003, *t*(178) = 3.500, *p* = 0.001, *Cohen’s d* = 0.262	*M* = 0.001, *SEM* = 0.03, *t*(178) = 0.233, *p* = 0.816, *Cohen’s d* = 0.018	*M* = 0.069, *SEM* = 0.004, *t*(179) = 16.136, *p* < 0.001, *Cohen’s d* = 1.202
	% of positive learners	53.33%	22.35%	14.53%	63.89%
	% of negative learners	3.33%	9.50%	12.29%	1.11%
	% of true learners	50.00%	12.85%	2.24%	62.78%

Lastly, we wanted to assess how these results relate to those obtained with the typical analysis methods (Model 1, No Filter; and Model 2 and Model 3, Triplet Filter). Figure B in S1 File shows mean effect sizes of various learning scores (obtained for reaction times); Figure C in S1 File shows the percentage of positive and negative learners (broken down by epochs) obtained for reaction times data; and **Figure D** in S1 File shows the percentage of positive and negative learners obtained for error data. We also identified reliably positive learners (based on reaction times) for earlier Models, and calculated Phi coefficients between pairs of learning scores of different Models to assess whether there is a correspondence of who is considered a reliable learner with the different analysis methods. The results showed that the correspondence is low (the highest Phi value being around 0.4; see Table P in S1 File.

## Discussion

The ASRT [4] task is a visuomotor sequence learning task designed to measure implicit learning and memory. In this paper, we discussed in detail the many possible information types that could be learned (such as pattern learning and different levels of joint frequency learning and conditional probability learning), and our concerns that these types of learning are not sufficiently differentiated by the currently used analysis methods. Moreover, as we have shown, the learning measures that are typically extracted from data might be biased by pre-existing tendencies to certain stimulus combinations, indicating that the ASRT does not measure (only) what it supposed to. We provided a presentation of how different analysis methods and filtering methods result in different levels of artifacts and biases, a hopefully practical aid for the (re)interpretation of the results obtained with the task. We also proposed new analysis methods (with a somewhat new terminology) and a filtering method that eliminates at least some of the biases discussed so far and thus can be used in future studies (or for reanalyzing already existing datasets).

### Are the new analysis methods better?

In the second section of the paper, we compared the goodness of fit of models that are the basis of the different analysis methods (models already in use, and those that we proposed in the current paper). Our results showed that more elaborate models have a better fit indicating that there is a benefit of using these (newly proposed) methods instead of the typically used ones. We also looked at the specific effects that could be extracted from data (more elaborate models having more and purer measures), and how different filtering methods alter the effect sizes of these measures on the individual level. Our result indicated that different filtering sometimes leads to both quantitatively and qualitatively different conclusions. Importantly, quad filter did not only affect the specific effect sizes but also increased the individual variability that could be detected with the task. And variability is crucial–if everyone seems to show the same performance, there is no need to measure it.

### What did the new analyses reveal?

With the usual triplet filter, we replicated the findings of Song et al. [46] and Németh et al. [17] who demonstrated that RH trials were (at least in the first few epochs of the task) reacted to faster and more accurately than PH trials. This paradoxical pattern of results still remained when H1 vs. H2 trials were compared instead of PH and RH trials–in the latter case, this could have been interpreted as a reflection of higher *joint probability learning* (contrasted with *conditional probability* learning) on the group level. However, using the quad filter, the paradoxical result disappeared, and even turned into its contrary (for a similar result see [46]), indicating that it was *conditional probability learning* that dominated. We here thus argue that the paradoxical pattern of results (RH performance > PH performance) is an artifact attributable to pre-existing biases on the quad level. This reasoning might be at odds with the fact that the paradoxical result was temporary in Song et. al [46]. However, Soetens, Boer, & Hueting [42] found that practice actually reduces sequential effects (i.e. pre-existing tendencies), while it is reasonable to assume that statistical learning would either stagnate or increase with practice.

This is an important finding since the terminology frequently found in ASRT studies implies joint frequency learning (e.g. the terms „low-frequency triplet”, „high-frequency triplet”, etc.) and not conditional probability learning. Such terminology might mislead researchers to have the wrong focus when complying stimulus material–even with a conservative viewpoint, both factors should be considered. Beside practical considerations, these results also add to the theoretical debate whether implicit learning is based on learning chunks (which in this case corresponds to joint probability learning, e.g. the relative frequency of different combinations) or statistical computations (conditional probability learning) [51].

Our new analysis methods revealed a dissociation between pattern learning and quad learning (which were confounded in previously quantified measures, i. e. *higher order learning*). On the group level, there was a significant quad learning effect (quantified as the difference between H1P and H2P trials), although smaller in magnitude than triplet level learning (quantified as the difference between H1R and LR trials); at the same time no pattern learning was observed (H1P vs. H1R) following this amount of practice. Moreover, H1P trials were slightly slower than H1R trials, which was unexpected. This new paradox might reflect pre-existing biases on the level of quints which were not controlled for in this study.

Besides these results observed on a group level, we also quantified the percentage of participants who showed at least a „small” learning effect (Cohen d > 0.2 in the cases of reaction times). From these percentages, we subtracted the percentage of participants showing similar effect sizes in the opposite directions (e.g. low probability trials being reacted to faster than high probability trials). The resulting number could be thought of as reflecting true learners (i. e. true positives without false positives). Strikingly, the percentage of participants showing at least a small triplet learning effect grew from ~25% to ~ 70% as learning progressed from epoch 1 to epoch 9 (interestingly, Parshina, Obeid, Che, Ricker, & Brooks [52], with a somewhat different methodology and using Model 2 as a basis of their analysis, found that 64.9% of their participants showed triplet learning on the ASRT task, corroborating the fact that approximately 1/3 of people fail to exhibit such learning). At the same time, the percentage of participants showing true pattern learning remained around zero throughout. Quad learning was observed for ~5% to ~20% of participants.There was fluctuation in these numbers but no gradual increase as learning progressed(a gradual increase could be observed in two out of three Sessions, though; see the Methods section. Although highly speculative, it is possible that at the beginning of Sessions participants are less tired, and their reaction times are faster and less variable, making it hard to detect such subtle effects). It is also possible that both joint frequency learning and trial probability learning occur, but since they have opposite effects on reaction times, it manifests as very low (near-zero) quad learning effect. The latter possibility should be assessed by a sequence learning task in which the two kinds of probabilities are systematically varied.

In the case of accuracy, small effect sizes were defined as a Cramer V value > 0.05. In this case, much smaller percentages of true learners were observed—the percentage of true pattern learners was ~2%, the percentage of true quad learners ~15% and the percentage of true triplet learners ~50%. Importantly, accuracy data has not provided information regarding the time course of learning.

It was interesting to see that quad learning was significant on the group level but the number of quad learners did not increase over the nine epochs. This result might indicate that the observed learning is some kind of artifact (e.g. preexisting biases that were not controlled for, e.g. on the level of quints), or that a few participants were able to extract quad level information from the very beginning of learning, while, for others, 45 blocks of ASRT was not enough for this. The latter interpretation is supported by data showing that higher order learning occurs slowly over many sessions, as in [4,46] although the differences seen in these studies are the mix of quad learning effects, pattern learning effects and pre-existing biases; while the support for the former interpretation comes from the fact that *pattern learning* was *negative* throughout the task which can only be attributed to pre-existing biases (since pattern trials have, on average, higher conditional probabilities/joint frequencies than random trials, thus they should be faster anyway). If such pre-existing biases affect the *pattern learning* measure this way, they could also be affecting the quad learning measure (but remember that quad learning is higher than pattern learning, irrespective of the sign, possibly suggesting true learning in addition to such biases). Future studies need to clarify the issue.

The finding that no detectable pattern learning occurred during these 45 blocks of ASRT is an important one, too. It suggests that the ASRT task is a primarily statistical learning task (and not a pattern learning one), at least after this amount of practice. If future research corroborates this finding even after extended practice with the task, then this measure could be used as an examination whether the hidden sequence remained undetected by participants, complementing the sorting tasks, e.g. [53], and production tasks, e.g. [54], usually utilized to assess explicit knowlegde. For explicit learners, i.e. who become aware of the hidden sequence and thus are able to consciously anticipate pattern trials but not random trials, this measure should have a positive value—as observed in the explicit variants of the ASRT task when assessing the *higher order learning* measure [17,46]. For implicit learners, on the other hand, the value of the measure should remain around zero.

Finally, we would like to draw attention to our way of quantifying individual learning as standardized effect sizes (Cohen’s d and Cramer’s V values) instead of just raw differences. This way the comparison of groups having different overall reaction times could be fairer; it’s easier to identify people who truly learned or at least the percentage of true learners (e.g. by selecting participants showing at least a small effect size throughout the task, e.g. Cohen’s d > 0.2). Due to increased individual variability, we could have missed the overall benefits of the newly proposed method looking only at group level effect sizes (e.g. when computing Cohen’s d, a higher difference score increases the effect size but higher variability decreases it), so it might have looked as if our method have no benefit over the typically used methods).

In sum, we believe that the analysis and filtering methods that we proposed have several advantages over the typically used methods. First, more types and more levels of learning can be detected, and thus it becomes possible to differentiate between people with similar overall learning scores but different learning profiles. Also, if two groups differ in overall scores, it becomes possible to disentangle which learning type causes the overall difference, and, in the long run, may help us build better models of implicit learning. Second, more strict filtering results in purer measures of learning and more expressed individual differences. The fact that individual variability is higher using the stricter filtering shows that some of the variability had been masked with typical filtering methods, which in turn questions the results showing weak or no correlation between statistical learning on the ASRT task and other measures, e.g. [5,52].

### Limitations and future directions

As a limitation for the newly proposed filtering method (but also for the typically used triplet filtering), it is important to note that these filters work on the group level when the six ASRT sequences are given to participants in the same proportions, but they do not necessarily work on the individual level (or when sequences are not counterbalanced across participants). The quads that are being compared within participants are similar in their abstract structure (consisting of *dcba*, *cbba* and *acba* quads), but some of the, say, *dcba* quads might still be easier than other exemplars of the same type. Also, as our results clearly indicated in the case of the pattern learning measure, the amount of biases may be modified even by the N-4^th^ trials (i.e. quint level pre-existing biases). Furthermore, the direction and magnitude of such biases may vary as a function of the ASRT sequence administered. Bearing this in mind, if the main focus of a study is measuring individual differences (rather than group differences and relatively bias-free average learning scores) than the same ASRT sequence should be given to all participants. Further studies should address which of the six sequences should be preferred (bearing the lowest amount of pre-existing biases), and they should clarify the relationship between sensitivity to such biases and learning capacity in the ASRT task (if there is any).

We only moved one step forward from the usual filtering–quad level information being considered on the group level instead of triplet level information. We decided to stop here, and not to go deeper, for two reasons. First, there are no quints that are part of each of the six ASRT sequences, thus full counterbalancing isn’t possible on the group level. Second, although some measures could be quantified in a more bias-free manner on the individual level if quint level information was considered, this would involve massive reduction in the number of trials being analyzed, and we speculated that the costs (increased noise) would be higher than the benefits (even smaller magnitude of pre-existing biases) of doing so.

Similarly, quint level statistical information could also have been considered but we decided not to go for it. The reason for this was that pre-existing biases could not have been ruled out even if we quantified this measure, and based on the small number of participants who actually learned quad level statistical information (~10–20%, see above), we speculated that quint level learning is probably close to zero after this amount of practice (thus this additional measure wouldn’t be of much use). On the other hand, such comparisons could only be made based on a smaller number of trials being analyzed, which could, in turn, result in increased noise.

The ASRT data analyzed in this paper consists of 45 blocks of 80 trials for each participant. Both shorter, e.g. [10], and longer, e.g. [4,16], ASRT-s have been used in the past, but it is reasonable to assume that *higher order learning*, i.e. quad learning and/or pattern learning occurs over longer periods of time; Methodsthe results of [4,46]. The analysis method proposed in this paper should be applied to data gained from extended practice to verify this hypothesis. This could reveal individual differences even in the dynamics of joint frequency vs. conditional probability learning, which would be an important step in understanding the nature of implicit statistical learning.

Future studies using longer ASRTs should clarify what types of learning are typical to humans and in what proportions. It should also be assessed whether these different types of learning correlate with each other and whether they rely on different brain structures (or on different kinds of connectivity/brain activity) [18].

## General conclusion

We believe that the ASRT task is a great tool for measuring implicit sequence learning and memory–it might even be more promising than we ever thought. However, in order to get more out of it, we need to improve our analysis methods and take possible confounding factors more seriously. In this paper, we provided a possible solution to these problems. Our results point to the ASRT being primarily a statistical learning task (at least in the short term), where triplet learning occurs for most of the participants but quad learning is the privilege of fewer. We have also shown that these results depend strongly on the filter being used, and for methodological reasons, we suggest the usage of the Quad Filter in the future.

Importantly, the concerns outlined in this work are not specific to the ASRT task. It is reasonable to assume that pre-existing biases are present in other sequence learning tasks as well, such as the serial reaction time task [2] or the finger tapping task [55]; and also that at least some people are sensitive to higher order statistics (e.g. third order statistical information), which should also be taken into consideration when complying stimulus material for all kinds of sequence learning tasks.

## Supporting information

S1 File Supplementary material. The file contains supplementary information cited in the manuscript as Figs A-D and Tables A-P.
**Fig A**. **Goodness of fit indicators (Adjusted R**^**2**^**s) of each Model (Model 1–5) and each filtering method (No Filter, Triplet Filter and Quad Filter) as a function of epochs (1–9).** Discontinuities of the lines indicate pauses between the three Sessions (Epochs 1–3, Epochs 4–6 and Epochs 7–9). Error bars represent the 95% CI.
**Fig B**. **Reaction time based learning scores calculated the typical way (M1 nofilter, M2 triplet filter and M3 triplet filter) and the proposed way (M5 quadfilter) in each of the nine epochs.** There was a longer pause between epochs 3 and 4, and between epochs 6 and 7 (creating three sessions, indicated by different colors). Bars represent the mean of the individual learning scores. Error bars represent 95% CI.
**Fig C**. **The percentage of participants showing learning based on reaction times with an effect size of Cohen’s d > 0.2, separately in each of the nine epochs.** Green lines represent participants whose learning scores were positive (i.e. the observed difference was in the expected direction). Black lines represent participants whose learning scores were negative (i.e. in the unexpected direction). The discontinuity of the lines indicate pauses during data collection (i.e. there were three sessions).
**Fig D**. **The percentage of participants showing learning based on error rates with an effect size of Cramer’s V > 0.05 (data of the nine epochs were collapsed into a single category due to low overall error rates).** Green bars represent participants whose learning scores were positive (i.e. the observed difference was in the expected direction). Black bars represent participants whose learning scores were negative (i.e. in the unexpected direction).
(DOCX)
