# Supplementary material for: Different levels of statistical learning - Hidden potentials of sequence learning tasks
Source: PLoS One. 2019 Sep 19;14(9):e0221966. doi: 10.1371/journal.pone.0221966 (PMC6752858; doi:10.1371/journal.pone.0221966)
Supplement: S1 File — The file contains supplementary information cited in the manuscript as Figs A-D and Tables A-P. Fig A. Goodness of fit indicators (Adjusted R2s) of each Model (Model 1–5) and each filtering method (No Filter, Triplet Filter and Quad Filter) as a function of epochs (1–9). Discontinuities of the lines indicate pauses between the three Sessions (Epochs 1–3, Epochs 4–6 and Epochs 7–9). Error bars represent the 95% CI. Fig B. Reaction time based learning scores calculated the typical way (M1 nofilter, M2 triplet filter and M3 triplet filter) and the proposed way (M5 quadfilter) in each of the nine epochs. There was a longer pause between epochs 3 and 4, and between epochs 6 and 7 (creating three sessions, indicated by different colors). Bars represent the mean of the individual learning scores. Error bars represent 95% CI. Fig C. The percentage of participants showing learning based on reaction times with an effect size of Cohen’s d > 0.2, separately in each of the nine epochs. Green lines represent participants whose learning scores were positive (i.e. the observed difference was in the expected direction). Black lines represent participants whose learning scores were negative (i.e. in the unexpected direction). The discontinuity of the lines indicate pauses during data collection (i.e. there were three sessions). Fig D. The percentage of participants showing learning based on error rates with an effect size of Cramer’s V > 0.05 (data of the nine epochs were collapsed into a single category due to low overall error rates). Green bars represent participants whose learning scores were positive (i.e. the observed difference was in the expected direction). Black bars represent participants whose learning scores were negative (i.e. in the unexpected direction). (DOCX) [file pone.0221966.s001.docx]

Supplementary Materials

Different levels of statistical learning - Hidden potentials of sequence learning tasks

Emese Szegedi-Hallgató^1,2,3^, Karolina Janacsek^4,5^, Dezso Nemeth^4,5,6 *^

^1^ Doctoral School of Psychology, ELTE Eötvös Loránd University, Budapest, Hungary

^2^ Institute of Psychology, Faculty of Humanities, University of Szeged, Szeged, Hungary

^3^ Prevention of Mental Illnesses Interdisciplinary Research Group, University of Szeged, Szeged, Hungary

^4^ Institute of Psychology, ELTE Eötvös Loránd University, Budapest, Hungary

^5^ Brain, Memory and Language Research Group, Institute of Cognitive Neuroscience and Psychology, Research Centre for Natural Sciences, Hungarian Academy of Sciences, Budapest, Hungary

^6^ Lyon Neuroscience Research Center, Université de Lyon, Lyon, France

* Corresponding author

Email: nemethd@gmail.com

**Description of Tables A-D**

Tables 5-8 supplement the Results (Section 1, including Fig 3-5) in the main text by providing supporting statistics.

**Table A** **Trial Type Proportions.** For each individual (N = 180) a Chi-Square test was run to assess whether random (R) and pattern (P) trials occur with the same relative frequency in the different categories present in a Model. Effect sizes (Cramer’s V) were also calculated individually. These computations were repeated using Triplet Filtering (TF) and Quad Filtering (QF). Values in the table represent the percentage of participants where the result of the Chi-Squared test was significant (*χ^2^ % participant significant*) and the mean Cramer’s V values and the standard deviation of these values (*Cramer’s V mean (SD)*).

**Table B** **Trial Probability**. To assess whether the distribution of trial probabilities (assessed either on triplet level or on quad level) were equal in the categories being contrasted for a particular learning score, Kolmogorov-Smirnov tests were run for each individual (N =180). To assess the direction of difference (if a difference was observed), an additional Mann-Whitney test was also run. Finally, the AUROC (Area Under the Reciever Operating Charachteristic Curve) was also calculated individually (AUC = Mann-Whitney U / (n_1_ x n_2_); where n_1_ and n_2_ refer to the two sample sizes). This statistics gives the probability that a randomly chosen value from one sample is higher that a randomly chosen value from the other sample, which we refer to as the *Probability of Superiority* in the Table.

The four values reported are then the following: The percentage of participants experiencing significantly different distributions of trial probabilities in the contrasted categories (*KS % participant significant*); the percentage of participants experiencing significantly higher trial probabilities in the first category (*MW % participant significant (a > b));* the percentage of participants experiencing significantly higher trial probabilities in the second category (*MW % participant significant (a > b)*), and the average probability that a randomly chosen member of category b is higher than a randomly chosen member of category A, along with the standard deviation of these values (*Probability of Superiority b > a mean % across participants (SD)*).

**Table C Combination Frequency.** The same statistics were calculated and presented that are described in *Table S2.*

**Table D The Abstract Structure of the Combinations**. The same statistics were calculated and presented as in *Table S1,* the only difference being that instead of trial proportions the relative frequency of abstact categories of combinations were compared.

**Table A. Trial Type Proportions.**

|  | | χ^2^  % participant significant | | | Cramer’s V  mean (*SD*) | | |
| --- | --- | --- | --- | --- | --- | --- | --- |
|  |  | NF | TF | QF | NF | TF | QF |
|  |  |  |  |  |  |  |  |
| M1 | R (a) vs. P (b)  Trial Type Effect | 100.00 | 100.00 | 100.00 | 1.00  *0.00* | 1.00  *0.00* | 1.00  *0.00* |
| M2 | L (a) vs. H (b)  Sequence Spec. L. | 100.00 | 100.00 | 100.00 | 0.78  *0.01* | 0.73  *0.01* | 0.58  *0.02* |
| M3 | LR (a) vs. HR (b)  Pure Statistical Learn. | 0.00 | 0.00 | 0.00 | 0.00  *0.00* | 0.00  *0.00* | 0.00  *0.00* |
|  | HR (a) vs. HP (b)  Higher Order Seq.L. | 100.00 | 100.00 | 100.00 | 1.00  *0.00* | 1.00  *0.00* | 1.00  *0.00* |
|  | LR (a) vs. HP (b)  Maximized Learning | 100.00 | 100.00 | 100.00 | 1.00  *0.00* | 1.00  *0.00* | 1.00  *0.00* |
| M4 | L (a) vs. H1 (b)  Triplet L. (+ P. L.) | 100.00 | 100.00 | 100.00 | 0.62  *0.00* | 0.58  *0.01* | 0.50  *0.01* |
|  | H1 (a) vs. H2 (b)  Quad L. (+ P. L.) | 100.00 | 100.00 | 100.00 | 0.61  *0.00* | 0.61  *0.00* | 0.50  *0.01* |
|  | L (a) vs. H2 (b)  Maximized Learning | 100.00 | 100.00 | 100.00 | 1.00  *0.00* | 1.00  *0.00* | 1.00  *0.00* |
| M5 | LR (a) vs. H1R (b)  Triplet Learning | 0.00 | 0.00 | 0.00 | 0.00  *0.00* | 0.00  *0.00* | 0.00  *0.00* |
|  | H1R (a) vs. H1P (b)  Pattern Learning | 100.00 | 100.00 | 100.00 | 1.00  *0.00* | 1.00  *0.00* | 1.00  *0.00* |
|  | H1P (a) vs. H2P (b)  Quad Learning | 0.00 | 0.00 | 0.00 | 0.00  *0.00* | 0.00  *0.00* | 0.00  *0.00* |
|  | LR (a) vs. H2P (b)  Maximized Learning | 100.00 | 100.00 | 100.00 | 1.00  *0.00* | 1.00  *0.00* | 1.00  *0.00* |

NF = No Filter, TF = Triplet Filter, QF = Quad Filter

**Table B. Combination Frequencies.**

|  | | Triplet Level | | | | | | | | | | | | Quad Level | | | | | | | | | | | |
| --- | --- | --- | --- | --- | --- | --- | --- | --- | --- | --- | --- | --- | --- | --- | --- | --- | --- | --- | --- | --- | --- | --- | --- | --- | --- |
|  |  | Kolmogorov-S.  % Participant significant | | | Mann-Whitney  % Participant significant | | | | | | Probability of Superiority  (b > a)  mean % across participants (*SD*) | | | Kolmogorov-S.  % Participant significant | | | Mann-Whitney  % Participant significant | | | | | | Probability of Superiority  (b > a)  mean % across participants (SD) | | |
|  |  | NF | TF | QF | NF | | TF | | QF | | NF | TF | QF | NF | TF | QF | NF | | TF | | QF | | NF | TF | QF |
|  |  |  |  |  | *a>b* | *b>a* | *a>b* | *a>b* | *b>a* | *a>b* |  |  |  |  |  |  | *a>b* | *b>a* | *a>b* | *b>a* | *a>b* | *b>a* |  |  |  |
| M1 | R (a) vs. P (b)  Trial Type Effect | 100 | 100 | 100 | 0.0 | 100 | 0.0 | 100 | 0.0 | 100 | 87.3 *0.6* | 83.1 *0.8* | 74.9 *1.3* | 31.7 | 100 | 50.6 | 0.0 | 5.6 | 91.1 | 0.0 | 4.4 | 17.2 | 51.3  *0.4* | 46.2  *1.1* | 50.8  *2.4* |
| M2 | L (a) vs. H (b)  Seq.Spec. L. | 100 | 100 | 100 | 0.0 | 100 | 0.0 | 100 | 0.0 | 100 | 99.7  *0.2* | 99.6  *0.2* | 99.6  *0.3* | 100 | 100 | 100 | 0.0 | 100 | 0.0 | 100 | 0.0 | 100 | 68.8  *1.3* | 67.2  *2.0* | 79.9  *3.7* |
| M3 | LR (a) vs. HR (b)  Pure Stat. Learn. | 100 | 100 | 100 | 0.0 | 100 | 0.0 | 100 | 0.0 | 100 | 99.7  *0.2* | 99.6  *0.3* | 99.6  *0.3* | 100 | 100 | 100 | 0.0 | 100 | 0.0 | 100 | 0.0 | 100 | 94.0  *2.0* | 93.5  *2.3* | 93.5  *2.8* |
|  | HR (a) vs. HP (b)  Higher O.Seq.L. | 3.9 | 3.9 | 2.8 | 1.7 | 1.7 | 1.7 | .17 | 0.6 | 2.2 | 50.1  *1.4* | 50.1  *1.4* | 50.1  *1.8* | 100 | 100 | 100 | 100 | 0.0 | 100 | 0.0 | 100 | 0.0 | 17.2  *1.5* | 17.2  *1.5* | 28.4  *1.8* |
|  | LR (a) vs. HP (b)  Maximized Learning | 100 | 100 | 100 | 0.0 | 100 | 0.0 | 100 | 0.0 | 100 | 99.6  *0.2* | 99.6  *0.3* | 99.6  *0.3* | 100 | 100 | 100 | 0.0 | 100 | 0.0 | 100 | 0.0 | 100 | 62.7  *1.0* | 60.8  *2.1* | 73.3  *4.7* |
| M4 | L (a) vs. H1 (b)  Triplet L.(+P. L.) | 100 | 100 | 100 | 0.0 | 100 | 0.0 | 100 | 0.0 | 100 | 99.7  *0.2* | 99.6  *0.3* | 99.6  *0.3* | 100 | 100 | 100 | 0.0 | 100 | 0.0 | 100 | 0.0 | 100 | 94.0  *2.0* | 93.4  *2.3* | 93.4  *2.7* |
|  | H1 (a) vs. H2 (b)  Quad L. (+ P. L.) | 8.9 | 8.9 | 7.2 | 3.9 | 5.6 | 3.9 | 5.6 | 1.1 | 3.3 | 50.3  *1.4* | 50.3  *1.4* | 50.4  *2.0* | 100 | 100 | 100 | 100 | 0.0 | 100 | 0.0 | 100 | 0.0 | 6.3  *2.1* | 6.3  *2.1* | 7.1  *3.1* |
|  | L (a) vs. H2 (b)  Maximized Learning | 100 | 100 | 100 | 0.0 | 100 | 0.0 | 100 | 0.0 | 100 | 99.6  *0.2* | 99.6  *0.3* | 99.6  *0.3* | 57.2 | 45.0 | 76.7 | 0.0 | 47.2 | 14.4 | 13.9 | 18.3 | 45.6 | 52.2  *0.6* | 49.9  *2.4* | 53.2  *8.4* |
| M5 | LR(a) vs. H1R(b)  Triplet Learning | 100 | 100 | 100 | 0.0 | 100 | 0.0 | 100 | 0.0 | 100 | 99.7  *0.2* | 99.6  *0.3* | 99.6  *0.3* | 100 | 100 | 100 | 0.0 | 100 | 0.0 | 100 | 0.0 | 100 | 94.0  *2.1* | 93.5  *2.3* | 93.5  *2.8* |
|  | H1R(a) vs. H1P(b)  Pattern Learning | 1.1 | 1.1 | 3.9 | 0.6 | 1.1 | 0.6 | 1.1 | 1.7 | 2.2 | 49.9  *1.4* | 49.9  *1.4* | 49.8  *2.1* | 1.1 | 1.1 | 1.7 | 0.6 | 0.0 | 0.6 | 0.0 | 0.6 | 0.6 | 49.8  *1.3* | 49.8  *1.3* | 49.8  *2.0* |
|  | H1P(a) vs. H2P(b)  Quad Learning | 4.4 | 4.4 | 8.3 | 1.1 | 3.9 | 1.1 | 3.9 | 2.8 | 4.4 | 50.3  *1.6* | 50.3  *1.6* | 50.5  *2.3* | 100 | 100 | 100 | 100 | 0.0 | 100 | 0.0 | 100 | 0.0 | 6.4  *2.1* | 6.4  *2.1* | 7.2  *3.2* |
|  | LR(a) vs. H2P(b)  Maximized Learning | 100 | 100 | 100 | 0.0 | 100 | 0.0 | 100 | 0.0 | 100 | 99.6  *0.2* | 99.6  *0.3* | 99.6  *0.3* | 57.2 | 45.0 | 76.7 | 0.0 | 47.2 | 14.4 | 13.9 | 18.3 | 45.6 | 52.2  *0.6* | 49.9  *2.4* | 53.2  *8.4* |

NF = No Filter, TF = Triplet Filter, QF = Quad Filter

**Table C. Conditional Probabilities (Trial Probabilities).**

|  | | Triplet Level | | | | | | | | | | | | Quad Level | | | | | | | | | | | |
| --- | --- | --- | --- | --- | --- | --- | --- | --- | --- | --- | --- | --- | --- | --- | --- | --- | --- | --- | --- | --- | --- | --- | --- | --- | --- |
|  |  | Kolmogorov-S.  % Participant significant | | | Mann-Whitney  % Participant significant | | | | | | Probability of Superiority  (b > a)  mean % across participants (*SD*) | | | Kolmogorov-S.  % Participant significant | | | Mann-Whitney  % Participant significant | | | | | | Probability of Superiority  (b > a)  mean % across participants (SD) | | |
|  |  | NF | TF | QF | NF | | TF | | QF | | NF | TF | QF | NF | TF | QF | NF | | TF | | QF | | NF | TF | QF |
|  |  |  |  |  | *a>b* | *b>a* | *a>b* | *a>b* | *b>a* | *a>b* |  |  |  |  |  |  | *a>b* | *b>a* | *a>b* | *b>a* | *a>b* | *b>a* |  |  |  |
| M1 | R (a) vs. P (b)  Trial Type Effect | 100 | 100 | 100 | 0.0 | 100 | 0.0 | 100 | 0.0 | 100 | 87.3  *0.6* | 83.1  *0.7* | 74.9  *1.3* | 100 | 100 | 100 | 0.0 | 100 | 0.0 | 100 | 0.0 | 100 | 94.3  *0.5* | 93.1  *0.6* | 84.6  *1.2* |
| M2 | L (a) vs. H (b)  Sequence Spec. L. | 100 | 100 | 100 | 0.0 | 100 | 0.0 | 100 | 0.0 | 100 | 99.7  *0.2* | 99.7  *0.2* | 99.7  *0.2* | 100 | 100 | 100 | 0.0 | 100 | 0.0 | 100 | 0.0 | 100 | 97.3  *0.8* | 97.0  *0.9* | 96.2  *1.7* |
| M3 | LR (a) vs. HR (b)  Pure Statistical Learn. | 100 | 100 | 100 | 0.0 | 100 | 0.0 | 100 | 0.0 | 100 | 99.7  *0.2* | 99.7  *0.2* | 99.7  *0.3* | 100 | 100 | 100 | 0.0 | 100 | 0.0 | 100 | 0.0 | 100 | 95.7  *1.8* | 95.1  *2.1* | 95.2  2.5 |
|  | HR (a) vs. HP (b)  Higher Order Seq.L. | 6.7 | 6.7 | 5.6 | 1.7 | 1.1 | 1.7 | 1.1 | 3.3 | 1.7 | 49.9  *1.5* | 49.9  *1.5* | 50.1  *2.0* | 100 | 100 | 100 | 0.0 | 100 | 0.0 | 100 | 0.0 | 100 | 84.4  *1.0* | 84.4  *1.0* | 72.6  *1.6* |
|  | LR (a) vs. HP (b)  Maximized Learning | 100 | 100 | 100 | 0.0 | 100 | 0.0 | 100 | 0.0 | 100 | 99.7  *0.2* | 99.7  *0.2* | 99.7  *0.2* | 100 | 100 | 100 | 0.0 | 100 | 0.0 | 100 | 0.0 | 100 | 97.7  *0.6* | 97.5  *0.7* | 96.6  *1.4* |
| M4 | L (a) vs. H1 (b)  Triplet L. (+ P. L.) | 100 | 100 | 100 | 0.0 | 100 | 0.0 | 100 | 0.0 | 100 | 99.8  *0.2* | 99.7  *0.2* | 99.7  *0.2* | 100 | 100 | 100 | 0.0 | 100 | 0.0 | 100 | 0.0 | 100 | 95.7  *1.9* | 95.1  *2.1* | 95.2  *2.6* |
|  | H1 (a) vs. H2 (b)  Quad L. (+ P. L.) | 3.3 | 3.3 | 2.8 | 2.8 | 1.1 | 2.8 | 1.1 | 0.6 | 4.4 | 50.0  *1.2* | 50.0  *1.2* | 50.6  *1.9* | 100 | 100 | 100 | 0.0 | 100 | 0.0 | 100 | 0.0 | 100 | 96.0  *0.9* | 96.0  *0.9* | 96.2  *1.6* |
|  | L (a) vs. H2 (b)  Maximized Learning | 100 | 100 | 100 | 0.0 | 100 | 0.0 | 100 | 0.0 | 100 | 99.7  *0.2* | 99.7  *0.2* | 99.7  *0.3* | 100 | 100 | 100 | 0.0 | 100 | 0.0 | 100 | 0.0 | 100 | 98.3  *0.4* | 98.3  *0.4* | 98.2  *0.7* |
| M5 | LR (a) vs. H1R (b)  Triplet Learning | 100 | 100 | 100 | 0.0 | 100 | 0.0 | 100 | 0.0 | 100 | 99.8  *0.2* | 99.7  *0.2* | 99.7  *0.3* | 100 | 100 | 100 | 0.0 | 100 | 0.0 | 100 | 0.0 | 100 | 95.7  *1.8* | 95.1  *2.1* | 95.2  *2.5* |
|  | H1R (a) vs. H1P (b)  Pattern Learning | 7.2 | 7.2 | 6.1 | 0.0 | 2.2 | 0.0 | 2.2 | 3.9 | 1.7 | 49.9  *1.9* | 49.9  *1.9* | 49.7  *2.3* | 0.6 | 0.6 | 1.1 | 0.6 | 1.7 | 0.6 | 1.7 | 1.7 | 0.6 | 49.9  *1.7* | 49.9  *1.7* | 49.8  *2.0* |
|  | H1P (a) vs. H2P (b)  Quad Learning | 2.8 | 2.8 | 4.4 | 0.6 | 2.8 | 0.6 | 2.8 | 1.1 | 6.1 | 50.1  *1.5* | 50.1  *1.5* | 50.7  *2.3* | 100 | 100 | 100 | 0.0 | 100 | 0.0 | 100 | 0.0 | 100 | 96.1  *0.8* | 96.1  *0.8* | 95.2  *1.6* |
|  | LR(a) vs. H2P(b)  Max.Learning | 100 | 100 | 100 | 0.0 | 100 | 0.0 | 100 | 0.0 | 100 | 99.7  *0.2* | 99.7  *0.2* | 99.7  *0.3* | 100 | 100 | 100 | 0.0 | 100 | 0.0 | 100 | 0.0 | 100 | 98.3  *0.4* | 94.3  *0.4* | 98.2  *0.6* |

NF = No Filter, TF = Triplet Filter, QF = Quad Filter

**Table D. Abstract Structure of the Combination.**

|  | | Triplet Level | | | | | | Quad Level | | | | | |
| --- | --- | --- | --- | --- | --- | --- | --- | --- | --- | --- | --- | --- | --- |
|  | | χ^2^  % participant significant | | | Cramer’s V  mean (*SD*) | | | χ^2^  % participant significant | | | Cramer’s V  mean (SD) | | |
|  |  | NF | TF | QF | NF | TF | QF | NF | TF | QF | NF | TF | QF |
|  |  |  |  |  |  |  |  |  |  |  |  |  |  |
| M1 | R (a) vs. P (b)  Trial Type Effect | 100.00 | 10.56 | 1.67 | 0.38  *0.01* | 0.03  *0.01* | 0.02  *0.01* | 100.00 | 100.00 | 7.22 | 0.50  *0.01* | 0.35  *0.01* | 0.04  *0.02* |
| M2 | L (a) vs. H (b)  Sequence Spec. L. | 100.00 | 3.89 | 4.44 | 0.49  *0.01* | 0.02  *0.01* | 0.02  *0.02* | 100.00 | 100.00 | 1.11 | 0.61  *0.01* | 0.41  *0.01* | 0.03  *0.02* |
| M3 | LR (a) vs. HP (b)  Pure Statistical Learn. | 100.00 | 6.11 | 5.56 | 0.34  *0.01* | 0.03  *0.02* | 0.03  *0.03* | 100.00 | 100.00 | 8.89 | 0.71  *0.01* | 0.66  *0.01* | 0.05  *0.03* |
|  | HR (a) vs. HP (b)  Higher Order Seq.L. | 11.11 | 11.11 | 6.11 | 0.03  *0.02* | 0.03  *0.02* | 0.03  *0.02* | 100.00 | 100.00 | 14.44 | 0.47  *0.01* | 0.47  *0.01* | 0.05  *0.03* |
|  | LR (a) vs. HP (b)  Maximized Learning | 100.00 | 5.56 | 2.78 | 0.47  *0.01* | 0.03  *0.01* | 0.02  *0.02* | 100.00 | 100.00 | 0.00 | 0.59  *0.01* | 0.40  *0.01* | 0.03  0.02 |
| M4 | L (a) vs. H1 (b)  Triplet L. (+ P. L.) | 100.00 | 7.78 | 6.11 | 0.41  *0.01* | 0.03  *0.02* | 0.03  *0.02* | 100.00 | 100.00 | 2.78 | 0.76  *0.01* | 0.71  *0.01* | 2.78  *0.02* |
|  | H1 (a) vs. H2 (b)  Quad L. (+ P. L.) | 5.00 | 5.00 | 5.56 | 0.02  *0.02* | 0.02  *0.02* | 0.03  *0.02* | 100.00 | 100.00 | 3.33 | 0.76  *0.01* | 0.76  *0.01* | 0.04  *0.02* |
|  | L (a) vs. H2 (b)  Maximized Learning | 100.00 | 2.22 | 2.22 | 0.45  *0.01* | 0.02  *0.01* | 0.03  *0.02* | 100.00 | 100.00 | 0.56 | 0.58  *0.01* | 0.41  *0.01* | 0.04  *0.02* |
| M5 | LR (a) vs. H1R (b)  Triplet Learning | 100.00 | 6.11 | 5.56 | 0.34  *0.01* | 0.03  *0.02* | 0.03  *0.03* | 100.00 | 100.00 | 8.89 | 0.71  *0.01* | 0.66  *0.01* | 0.05  *0.03* |
|  | H1R (a) vs. H1P (b)  Pattern Learning | 15.00 | 15.00 | 5.00 | 0.05  *0.03* | 0.05  *0.03* | 5.00  *0.02* | 26.11 | 26.11 | 19.44 | 0.07  *0.04* | 0.07  *0.04* | 0.07  *0.04* |
|  | H1P (a) vs. H2P (b)  Quad Learning | 5.56 | 5.56 | 4.44 | 0.03  *0.02* | 0.03  *0.02* | 0.03  *0.02* | 100.00 | 100.00 | 6.67 | 0.71  *0.01* | 0.71  *0.01* | 0.05  *0.03* |
|  | LR (a) vs. H2P (b)  Maximized Learning | 100.00 | 2.22 | 2.22 | 0.45  *0.01* | 0.02  *0.01* | 0.03  *0.02* | 100.00 | 100.00 | 0.56 | 0.58  *0.01* | 0.41  *0.01* | 0.04  0.02 |

NF = No Filter, TF = Triplet Filter, QF = Quad Filter

**Description of Tables E-O**

**Table E** Mean reaction times were calculated for each Models’ each subcategory in each epoch for each participant. Then the nine values belonging to the nine epochs were averaged yielding a single value per participant (RT mean). The Standard Deviation (RT SD) and Coefficient of Variation (RT CV, SD/mean, %) was also computed based on the spread of these means. Similarly, error percentages were calculated for each Models’ each subcategory in each epoch for each participant. Then the nine values belonging to the nine epochs were averaged yielding a single value per participant (Error %). The Standard Deviation (Error SD) and Coefficient of Variation (Error CV) were then computed based on the spread of these averages. All of these calculations were done for all three filtering types (NF = No Filter, TF = Triplet Filter, QF = Quad Filter).

**Table F** Learning scores were calculated for each Model. Learning scores based on reaction times are Cohen’s d values, computed separately for each individual and each epoch, and the nine values corresponding to the nine epochs were averaged to yield a single value for each participant (d mean). The spread of these means were quantified as Standard Deviations (d SD) and Coefficients of Variation (d CV).

In the case of error rates, data from the nine epochs was collapsed into a single category due to the low overall error rates. Cramer’s V values were computed for each Models’ each learning score individually, and the mean of these means is presented in the table (V mean). The spread of these means were also assessed (V SD and V CV).

All of these calculations were done for all three filtering types (NF = No Filter, TF = Triplet Filter, QF = Quad Filter).

**Tables G-L** In these tables the same descriptive statistics are shown as in **Table S6** (each new table corresponding to the subsequent row in Table 7), but here they are broken down by the ASRT sequence being used (the notations P1-P6 referring to the six pattern types).

**Table M** Within-subject variability of reaction times were quantified as Standard Deviations (SD mean (ms)) and as Coefficients of Variation (CV mean (%)). These descriptive statistics were computed for each Models’ each subcategory in each epoch for each participant. Then the nine values belonging to the nine epochs were averaged yielding a single value per participant. All of these calculations were done for all three filtering types (NF = No Filter, TF = Triplet Filter, QF = Quad Filter).

**Tables N-O** In these tables the same descriptive statistics are shown as in **Table S13** (separate tables corresponding to SDs and CVs), but here the statistics are broken down by the ASRT sequence being used (the notations P1-P6 referring to the six pattern types).

**Table E. Descriptive statistics of mean reaction times and error percentages with standard deviations and coefficients of variations for each Models’ each subcategory.**

|  | | Model 1 | | Model 2 | | Model 3 | | | Model 4 | | | Model 5 | | | |
| --- | --- | --- | --- | --- | --- | --- | --- | --- | --- | --- | --- | --- | --- | --- | --- |
|  |  | P | R | H | L | HP | HR | LR | H2 | H1 | L | H2P | H1P | H1R | LR |
| RT mean | NF | 373.59 | 381.18 | 372.07 | 386.51 | 373.59 | 365.92 | 386.51 | 375.72 | 366.77 | 386.51 | 375.72 | 367.57 | 365.92 | 386.51 |
|  | TF | 373.59 | 379.14 | 372.07 | 385.99 | 373.59 | 365.92 | 385.99 | 375.72 | 366.77 | 385.99 | 375.72 | 367.57 | 365.92 | 385.99 |
|  | QF | 377.32 | 385.83 | 377.43 | 394.28 | 377.32 | 377.57 | 394.28 | 375.74 | 378.29 | 394.28 | 375.74 | 378.90 | 377.57 | 394.28 |
| RT SD | NF | 27.80 | 28.39 | 27.73 | 29.00 | 27.80 | 27.81 | 29.00 | 27.99 | 27.64 | 29.00 | 27.99 | 27.66 | 27.81 | 29.00 |
|  | TF | 27.80 | 28.24 | 27.73 | 28.93 | 27.80 | 27.81 | 28.93 | 27.99 | 27.64 | 28.93 | 27.99 | 27.66 | 27.81 | 28.93 |
|  | QF | 29.96 | 31.52 | 30.30 | 32.58 | 29.96 | 31.36 | 32.58 | 29.79 | 30.83 | 32.58 | 29.79 | 30.56 | 31.36 | 32.58 |
| RT CV | NF | 7.44 | 7.45 | 7.45 | 7.50 | 7.44 | 7.60 | 7.50 | 7.45 | 7.54 | 7.50 | 7.45 | 7.53 | 7.60 | 7.50 |
|  | TF | 7.44 | 7.45 | 7.45 | 7.49 | 7.44 | 7.60 | 7.50 | 7.45 | 7.54 | 7.49 | 7.45 | 7.53 | 7.60 | 7.49 |
|  | QF | 7.94 | 8.17 | 8.03 | 8.26 | 7.94 | 8.31 | 8.26 | 7.93 | 8.15 | 8.26 | 7.93 | 8.07 | 8.31 | 8.26 |
| Error (%) | NF | 3.95 | 6.15 | 3.89 | 6.99 | 3.95 | 3.65 | 6.99 | 4.05 | 3.70 | 6.99 | 4.05 | 3.69 | 3.65 | 6.99 |
|  | TF | 3.95 | 5.60 | 3.89 | 6.59 | 3.95 | 3.65 | 6.59 | 4.05 | 3.70 | 6.59 | 4.05 | 3.69 | 3.65 | 6.59 |
|  | QF | 4.45 | 6.17 | 4.57 | 7.56 | 4.45 | 4.77 | 7.56 | 4.15 | 4.78 | 7.56 | 4.15 | 4.75 | 4.77 | 7.56 |
| Error SD | NF | 2.68 | 3.50 | 2.60 | 3.97 | 2.68 | 2.51 | 3.97 | 2.80 | 2.48 | 3.97 | 2.80 | 2.63 | 2.51 | 3.97 |
|  | TF | 2.68 | 3.29 | 2.60 | 3.89 | 2.68 | 2.51 | 3.89 | 2.80 | 2.48 | 3.89 | 2.80 | 2.63 | 2.51 | 3.89 |
|  | QF | 3.01 | 3.73 | 3.04 | 4.70 | 3.01 | 3.35 | 4.70 | 2.94 | 3.26 | 4.70 | 2.94 | 3.42 | 3.35 | 4.70 |
| Error CV | NF | 67.89 | 56.91 | 66.68 | 56.81 | 67.89 | 68.72 | 56.81 | 69.05 | 67.08 | 56.81 | 69.05 | 71.43 | 68.72 | 56.81 |
|  | TF | 67.89 | 58.71 | 66.68 | 58.96 | 67.89 | 68.72 | 58.96 | 69.05 | 67.08 | 58.96 | 69.05 | 71.43 | 68.72 | 58.96 |
|  | QF | 67.73 | 60.49 | 66.51 | 62.12 | 67.73 | 70.28 | 62.12 | 70.76 | 68.20 | 62.12 | 70.76 | 71.96 | 70.28 | 62.12 |

RT = reaction times, SD = standard deviation, CV = Coefficient of Variation (SD/mean, %), NF = No Filter, TF = Triplet Filter, QF = Quad Filter

**Table F. Mean individual effect sizes of learning, SD of these effect sizes and the CV of these effect sizes.**

|  | | | Model 1 | Model 2 | Model 3 | | | | | Model 4 | | | Model 5 | | | |
| --- | --- | --- | --- | --- | --- | --- | --- | --- | --- | --- | --- | --- | --- | --- | --- | --- |
|  |  |  | R-P | L-H | HR-HP | | LR-HR | LR-HP | | H1-H2 | L-H1 | L-H2 | H1P-H2P | H1R-H1P | LR-H1R | LR-H2P |
| d mean | NF | | .153 | .283 | -.153 | | .407 | .256 | | -.174 | .385 | .217 | -.164 | -.032 | .407 | .217 |
|  | TF | | .113 | .272 | -.153 | | .396 | .244 | | -.174 | .375 | .206 | -.164 | -.032 | .396 | .206 |
|  | QF | | .172 | .340 | -.004 | | .350 | .343 | | .047 | .328 | .376 | .056 | -.028 | .350 | .376 |
| d  SD | NF | | .106 | .136 | .093 | | .156 | .137 | | .100 | .150 | .141 | .112 | .089 | .156 | .141 |
|  | TF | | .082 | .115 | .093 | | .147 | .114 | | .100 | .138 | .117 | .112 | .089 | .147 | .117 |
|  | QF | | .100 | .171 | .104 | | .192 | .170 | | .121 | .180 | .182 | .134 | .116 | .192 | .182 |
| d  CV | | NF | 69.36 | 47.95 | | 60.41 | 38.41 | 53.51 | 57.44 | | 38.95 | 64.97 | 68.57 | 276.19 | 38.41 | 64.97 |
|  |  | TF | 72.62 | 42.31 | | 60.41 | 37.09 | 46.73 | 57.44 | | 36.73 | 56.91 | 68.57 | 276.19 | 37.09 | 56.91 |
|  |  | QF | 57.90 | 50.15 | | 2543.49 | 54.87 | 49.65 | 265.81 | | 54.86 | 48.41 | 241.79 | 409.74 | 54.87 | 48.41 |
| V mean | | NF | .050 | .068 | | -.005 | .059 | .067 | -.009 | | .069 | .064 | -.008 | .000 | .059 | .064 |
|  |  | TF | .039 | .058 | | -.005 | .058 | .058 | -.009 | | .065 | .056 | -.008 | .000 | .058 | .056 |
|  |  | QF | .037 | .058 | | .006 | .057 | .063 | .012 | | .057 | .069 | .012 | .001 | .057 | .069 |
| V  SD | | NF | .031 | .036 | | .026 | .031 | .037 | .028 | | .036 | .036 | .029 | .038 | .031 | .036 |
|  |  | TF | .029 | .034 | | .026 | .037 | .035 | .028 | | .038 | .036 | .029 | .038 | .037 | .036 |
|  |  | QF | .037 | .049 | | .036 | .055 | .053 | .037 | | .051 | .058 | .047 | .045 | .055 | .058 |
| V  CV | | NF | 61.55 | 52.37 | | -468.25 | 53.58 | 54.69 | -306.23 | | 51.74 | 56.95 | -358.67 | -30905.4 | 53.58 | 56.95 |
|  |  | TF | 73.28 | 58.70 | | -468.25 | 62.50 | 60.94 | -306.23 | | 58.36 | 64.32 | -358.67 | -30905.4 | 62.50 | 64.32 |
|  |  | QF | 98.50 | 84.58 | | 581.77 | 97.46 | 83.45 | 311.89 | | 90.50 | 83.15 | 382.23 | 5738.97 | 97.46 | 83.15 |

RT = reaction times, SD = standard deviation, CV = Coefficient of Variation (SD/mean, %), NF = No Filter, TF = Triplet Filter, QF = Quad Filter

**Table G. Mean individual effect sizes (Cohen’s d) computed for each Models’ learning scores broken down by the ASRT sequences being taught (P1-P6). Effect sizes are derived from reaction times.**

|  | | Model 1 | Model 2 | Model 3 | | | Model 4 | | | Model 5 | | | |
| --- | --- | --- | --- | --- | --- | --- | --- | --- | --- | --- | --- | --- | --- |
|  |  | R-P | L-H | HR-HP | LR-HR | LR-HP | H1-H2 | L-H1 | L-H2 | H1P-H2P | H1R-H1P | LR-H1R | LR-H2P |
| P1 | NF | .153 | .297 | -.178 | .438 | .265 | -.190 | .407 | .226 | -.168 | -.055 | .438 | .226 |
|  | TF | .110 | .285 | -.178 | .427 | .253 | -.190 | .396 | .213 | -.168 | -.055 | .427 | .213 |
|  | QF | .133 | .320 | -.049 | .357 | .309 | -.110 | .326 | .316 | .013 | -.055 | .357 | .316 |
| P2 | NF | .223 | .375 | -.146 | .493 | .348 | -.159 | .466 | .315 | -.143 | -.037 | .493 | .315 |
|  | TF | .164 | .346 | -.146 | .465 | .319 | -.159 | .438 | .286 | -.143 | -.037 | .465 | .286 |
|  | QF | .234 | .467 | .006 | .473 | .468 | .106 | .434 | .538 | .130 | -.057 | .473 | .538 |
| P3 | NF | .123 | .243 | -.152 | .362 | .216 | -.212 | .366 | .163 | -.226 | .011 | .362 | .163 |
|  | TF | .093 | .242 | -.152 | .362 | .215 | -.212 | .365 | .161 | -.226 | .011 | .362 | .161 |
|  | QF | .189 | .318 | .038 | .294 | .337 | .018 | .313 | .342 | -.011 | .048 | .294 | .342 |
| P4 | NF | .141 | .257 | -.139 | .372 | .233 | -.136 | .339 | .206 | -.114 | -.053 | .372 | .206 |
|  | TF | .108 | .251 | -.139 | .365 | .227 | -.136 | .333 | .200 | -.114 | -.053 | .365 | .200 |
|  | QF | .173 | .330 | .002 | .340 | .333 | .102 | .302 | .396 | .124 | -.058 | .340 | .396 |
| P5 | NF | .124 | .238 | -.144 | .354 | .212 | -.186 | .348 | .166 | -.189 | -.006 | .354 | .166 |
|  | TF | .079 | .214 | -.144 | .330 | .188 | -.186 | .324 | .142 | -.189 | -.006 | .330 | .142 |
|  | QF | .156 | .277 | .015 | .271 | .287 | .040 | .267 | .311 | .034 | .004 | .271 | .311 |
| P6 | NF | .158 | .294 | -.162 | .428 | .266 | -.164 | .393 | .234 | -.144 | –.052 | .428 | .234 |
|  | TF | .128 | .300 | -.162 | .435 | .271 | -.164 | .399 | .240 | -.144 | -.052 | .435 | .240 |
|  | QF | .150 | .334 | -.039 | .371 | .326 | .024 | .333 | .355 | .041 | -.055 | .371 | .355 |

P1-P6 = Pattern1-Pattern6, NF = No Filter, TF = Triplet Filter, QF = Quad Filter

**Table H. Individual effect size (Cohen’s d) SDs computed for each Models’ learning scores broken down by the ASRT sequences being taught (P1-P6). Effect sizes are derived from reaction times.**

|  | | Model 1 | Model 2 | Model 3 | | | Model 4 | | | Model 5 | | | |
| --- | --- | --- | --- | --- | --- | --- | --- | --- | --- | --- | --- | --- | --- |
|  |  | R-P | L-H | HR-HP | LR-HR | LR-HP | H1-H2 | L-H1 | L-H2 | H1P-H2P | H1R-H1P | LR-H1R | LR-H2P |
| P1 | NF | .109 | .134 | .092 | .138 | .138 | .104 | .137 | .145 | .111 | .074 | .138 | .145 |
|  | TF | .082 | .107 | .092 | .132 | .108 | .104 | .131 | .111 | .111 | .074 | .132 | .111 |
|  | QF | .105 | .173 | .099 | .180 | .180 | .136 | .174 | .211 | .144 | .091 | .180 | .211 |
| P2 | NF | .093 | .128 | .064 | .145 | .126 | .077 | .150 | .120 | .094 | .071 | .145 | .120 |
|  | TF | .069 | .111 | .064 | .138 | .108 | .077 | .134 | .106 | .094 | .071 | .138 | .106 |
|  | QF | .092 | .151 | .099 | .173 | .152 | .113 | .169 | .143 | .129 | .118 | .173 | .143 |
| P3 | NF | .090 | .134 | .103 | .186 | .128 | .107 | .174 | .124 | .115 | .099 | .186 | .124 |
|  | TF | .066 | .110 | .103 | .167 | .103 | .107 | .153 | .097 | .115 | .099 | .167 | .097 |
|  | QF | .090 | .160 | .116 | .201 | .150 | .116 | .183 | .145 | .127 | .132 | .201 | .145 |
| P4 | NF | .104 | .121 | .084 | .132 | .126 | .084 | .123 | .133 | .099 | .093 | .132 | .133 |
|  | TF | .082 | .103 | .084 | .126 | .105 | .084 | .120 | .107 | .099 | .093 | .126 | .107 |
|  | QF | .100 | .152 | .089 | .161 | .158 | .094 | .156 | .161 | .115 | .115 | .161 | .161 |
| P5 | NF | .099 | .107 | .115 | .128 | .114 | .121 | .119 | .128 | .122 | .084 | .128 | .128 |
|  | TF | .081 | .087 | .115 | .126 | .092 | .121 | .114 | .102 | .122 | .084 | .126 | .102 |
|  | QF | .070 | .144 | .101 | .185 | .130 | .118 | .166 | .129 | .112 | .094 | .185 | .129 |
| P6 | NF | .116 | .151 | .091 | .165 | .153 | .090 | .167 | .151 | .102 | .091 | .165 | .151 |
|  | TF | .090 | .130 | .091 | .155 | .130 | .090 | .149 | .131 | .102 | .091 | .155 | .131 |
|  | QF | .112 | .194 | .098 | .201 | .199 | .105 | .205 | .199 | .116 | .107 | .201 | .199 |

P1-P6 = Pattern1-Pattern6, NF = No Filter, TF = Triplet Filter, QF = Quad Filter

**Table I. Individual effect size (Cohen’s d) CVs computed for each Models’ learning scores broken down by the ASRT sequences being taught (P1-P6). Effect sizes are derived from reaction times.**

|  | | Model 1 | Model 2 | Model 3 | | | Model 4 | | | Model 5 | | | |
| --- | --- | --- | --- | --- | --- | --- | --- | --- | --- | --- | --- | --- | --- |
|  |  | R-P | L-H | HR-HP | LR-HR | LR-HP | H1-H2 | L-H1 | L-H2 | H1P-H2P | H1R-H1P | LR-H1R | LR-H2P |
| P1 | NF | 71.07 | 45.00 | 51.65 | 31.59 | 51.89 | 55.05 | 33.77 | 63.97 | 66.53 | 134.01 | 31.59 | 63.97 |
|  | TF | 74.12 | 37.65 | 51.65 | 30.88 | 42.83 | 55.05 | 32.98 | 51.92 | 66.53 | 134.01 | 30.88 | 51.92 |
|  | QF | 78.93 | 54.17 | 201.13 | 50.46 | 58.21 | 1370.95 | 53.25 | 66.65 | 1106.34 | 166.04 | 50.46 | 66.65 |
| P2 | NF | 41.87 | 34.07 | 44.25 | 29.05 | 36.21 | 48.09 | 32.22 | 38.22 | 65.38 | 193.63 | 29.50 | 38.22 |
|  | TF | 41.79 | 32.19 | 44.25 | 29.61 | 33.78 | 48.09 | 30.52 | 36.90 | 65.38 | 193.63 | 29.61 | 36.90 |
|  | QF | 39.25 | 32.39 | 1532.23 | 36.58 | 32.55 | 106.32 | 38.92 | 26.53 | 98.98 | 206.21 | 36.58 | 26.53 |
| P3 | NF | 73.29 | 55.24 | 67.94 | 51.37 | 59.18 | 50.36 | 47.54 | 76.00 | 51.03 | 895.22 | 51.37 | 76.00 |
|  | TF | 71.05 | 45.55 | 67.94 | 46.15 | 47.88 | 50.36 | 42.07 | 59.99 | 51.03 | 895.22 | 46.15 | 59.99 |
|  | QF | 47.36 | 50.49 | 307.93 | 68.34 | 44.53 | 684.08 | 58.46 | 42.24 | 1163.74 | 273.37 | 68.34 | 42.24 |
| P4 | NF | 74.18 | 47.15 | 60.57 | 35.66 | 54.12 | 62.01 | 36.28 | 64.61 | 86.36 | 173.94 | 35.66 | 64.61 |
|  | TF | 76.66 | 41.14 | 60.57 | 34.59 | 46.52 | 62.01 | 36.03 | 53.25 | 86.36 | 173.94 | 34.59 | 53.25 |
|  | QF | 57.92 | 45.94 | 4440.50 | 47.40 | 47.49 | 91.45 | 51.65 | 40.74 | 92.82 | 198.34 | 47.40 | 40.74 |
| P5 | NF | 80-39 | 45.17 | 80.20 | 36.26 | 53.86 | 65.05 | 34.17 | 77.18 | 64.87 | 1413.96 | 36.26 | 77.18 |
|  | TF | 102.48 | 40.80 | 80.20 | 38.07 | 48.82 | 65.05 | 35.03 | 72.30 | 64.87 | 1413.96 | 38.07 | 72.30 |
|  | QF | 45.19 | 51.89 | 655.02 | 68.29 | 45.29 | 293.38 | 62.21 | 41.63 | 328.69 | 2205.81 | 68.29 | 41.63 |
| P6 | NF | 72.93 | 51.27 | 55.98 | 38.51 | 57.40 | 54.91 | 42.62 | 64.51 | 70.84 | 174.94 | 38.51 | 64.51 |
|  | TF | 70.70 | 43.23 | 55.98 | 35.58 | 48.00 | 54.91 | 37.31 | 54.51 | 70.84 | 174.94 | 35.58 | 54.51 |
|  | QF | 74.74 | 57.98 | 248.52 | 54.35 | 60.95 | 429.84 | 61.61 | 56.11 | 282.94 | 196.45 | 54.35 | 56.11 |

P1-P6 = Pattern1-Pattern6, NF = No Filter, TF = Triplet Filter, QF = Quad Filter

**Table J. Mean individual effect sizes (Cramer’s V) computed for each Models’ learning scores broken down by the ASRT sequences being taught (P1-P6). Effect sizes are derived from error rates**

|  | | Model 1 | Model 2 | Model 3 | | | Model 4 | | | Model 5 | | | |
| --- | --- | --- | --- | --- | --- | --- | --- | --- | --- | --- | --- | --- | --- |
|  |  | R-P | L-H | HR-HP | LR-HR | LR-HP | H1-H2 | L-H1 | L-H2 | H1P-H2P | H1R-H1P | LR-H1R | LR-H2P |
| P1 | NF | .047 | .066 | -.009 | .060 | .064 | -.020 | .074 | .057 | -.020 | .006 | .060 | .057 |
|  | TF | .035 | .054 | -.009 | .059 | .054 | -.020 | .069 | .049 | -.020 | .006 | .059 | .049 |
|  | QF | .038 | .058 | .007 | .057 | .064 | .002 | .061 | .063 | -.002 | .009 | .057 | .063 |
| P2 | NF | .054 | .073 | -.006 | .062 | .072 | -.008 | .073 | .069 | -.006 | -.004 | .062 | .069 |
|  | TF | .043 | .063 | -.006 | .063 | .063 | -.008 | .070 | .062 | -.006 | -.004 | .063 | .062 |
|  | QF | .045 | .073 | .003 | .075 | .078 | .014 | .072 | .087 | .017 | -.005 | .075 | .087 |
| P3 | NF | .048 | .063 | -.002 | .052 | .063 | -.004 | .062 | .061 | -.005 | .003 | .052 | .061 |
|  | TF | .040 | .056 | -.002 | .053 | .057 | -.004 | .060 | .057 | -.005 | .003 | .053 | .057 |
|  | QF | .029 | .039 | .010 | .034 | .044 | .017 | .036 | .054 | .017 | .004 | .034 | .054 |
| P4 | NF | .058 | .078 | -.005 | .065 | .077 | .000 | .073 | .076 | .004 | -.011 | .065 | .076 |
|  | TF | .047 | .068 | -.005 | .066 | .069 | .000 | .069 | .071 | .004 | -.011 | .066 | .071 |
|  | QF | .043 | .072 | .001 | .074 | .076 | .018 | .069 | .087 | .025 | -.011 | .074 | .087 |
| P5 | NF | .048 | .065 | -.004 | .056 | .064 | -.010 | .067 | .060 | -.011 | .004 | .056 | .060 |
|  | TF | .039 | .057 | -.004 | .058 | .057 | -.010 | .065 | .055 | -.011 | .004 | .058 | .055 |
|  | QF | .041 | .058 | .013 | .054 | .066 | .018 | .055 | .075 | .016 | .006 | .054 | .075 |
| P6 | NF | .044 | .062 | -.007 | .055 | .060 | -.013 | .066 | .056 | -.012 | .002 | .055 | .056 |
|  | TF | .029 | .045 | -.007 | .049 | .048 | -.013 | .055 | -.043 | -.012 | .002 | .049 | .043 |
|  | QF | .026 | .043 | .003 | .045 | .047 | .001 | .045 | .047 | -.001 | .003 | .045 | .047 |

P1-P6 = Pattern1-Pattern6, NF = No Filter, TF = Triplet Filter, QF = Quad Filter

**Table K. Individual effect size (Cramer’s V) SDs computed for each Models’ learning scores broken down by the ASRT sequences being taught (P1-P6). Effect sizes are derived from error rates.**

|  | | Model 1 | Model 2 | Model 3 | | | Model 4 | | | Model 5 | | | |
| --- | --- | --- | --- | --- | --- | --- | --- | --- | --- | --- | --- | --- | --- |
|  |  | R-P | L-H | HR-HP | LR-HR | LR-HP | H1-H2 | L-H1 | L-H2 | H1P-H2P | H1R-H1P | LR-H1R | LR-H2P |
| P1 | NF | .034 | .036 | .026 | .028 | .039 | .029 | .033 | .039 | .027 | .035 | .028 | .039 |
|  | TF | .030 | .030 | .026 | .029 | .033 | .029 | .032 | .034 | .027 | .035 | .029 | .034 |
|  | QF | .043 | .055 | .038 | .059 | .061 | .038 | .057 | .065 | .045 | .044 | .059 | .065 |
| P2 | NF | .025 | .033 | .027 | .035 | .031 | .027 | .037 | .029 | .028 | .041 | .035 | .029 |
|  | TF | .023 | .034 | .027 | .044 | .033 | .027 | .042 | .032 | .028 | .041 | .044 | .032 |
|  | QF | .033 | .050 | .038 | .060 | .051 | .035 | .054 | .053 | .043 | .044 | .060 | .053 |
| P3 | NF | .036 | .044 | .025 | .037 | .045 | .031 | .043 | .045 | .033 | .037 | .037 | .045 |
|  | TF | .032 | .040 | .025 | .042 | .042 | .031 | .045 | .044 | .033 | .037 | .042 | .044 |
|  | QF | .026 | .039 | .030 | .050 | .040 | .044 | .045 | .049 | .058 | .042 | .050 | .049 |
| P4 | NF | .026 | .030 | .025 | .028 | .030 | .021 | .027 | .030 | .021 | .036 | .028 | .030 |
|  | TF | .026 | .032 | .025 | .035 | .032 | .021 | .033 | .034 | .021 | .036 | .035 | .034 |
|  | QF | .039 | .052 | .037 | .058 | .056 | .031 | .052 | .062 | .037 | .044 | .058 | .062 |
| P5 | NF | .029 | .033 | .029 | .033 | .034 | .031 | .038 | .032 | .030 | .038 | .033 | .032 |
|  | TF | .028 | .030 | .029 | .037 | .032 | .031 | .039 | .032 | .030 | .038 | .037 | .032 |
|  | QF | .032 | .041 | .032 | .047 | .044 | .036 | .046 | .046 | .051 | .044 | .047 | .046 |
| P6 | NF | .033 | .037 | .023 | .028 | .039 | .025 | .036 | .038 | .031 | .044 | .028 | .038 |
|  | TF | .031 | .034 | .023 | .031 | .037 | .025 | .037 | .037 | .031 | .044 | .031 | .037 |
|  | QF | .044 | .045 | .041 | .046 | .055 | .032 | .046 | .058 | .041 | .051 | .046 | .058 |

SD = Standard Deviation, P1-P6 = Pattern1-Pattern6, NF = No Filter, TF = Triplet Filter, QF = Quad Filter

**Table L. Individual effect size (Cramer’s V) CVs computed for each Models’ learning scores broken down by the ASRT sequences being taught (P1-P6). Effect sizes are derived from error rates.**

|  | | Model 1 | Model 2 | Model 3 | | | Model 4 | | | Model 5 | | | |
| --- | --- | --- | --- | --- | --- | --- | --- | --- | --- | --- | --- | --- | --- |
|  |  | R-P | L-H | HR-HP | LR-HR | LR-HP | H1-H2 | L-H1 | L-H2 | H1P-H2P | H1R-H1P | LR-H1R | LR-H2P |
| P1 | NF | 72.80 | 55.00 | -291.30 | 45.84 | 60.81 | -142.75 | 44.91 | 68.33 | -130.71 | 621.41 | 45.84 | 68.33 |
|  | TF | 85.58 | 55.59 | -291.30 | 48.85 | 62.06 | -142.75 | 46.51 | 70.75 | -130.71 | 621.41 | 58.85 | 70.75 |
|  | QF | 112.75 | 94.26 | 540.84 | 103.45 | 95.07 | 2349.56 | 93.89 | 103.91 | -2026.90 | 507.06 | 103.45 | 103.91 |
| P2 | NF | 46.27 | 44.85 | -411.41 | 55.70 | 43.57 | -325.35 | 51.15 | 41.96 | -462.69 | -1117.24 | 55.70 | 41.96 |
|  | TF | 53.95 | 54.31 | -411.41 | 69.01 | 51.67 | -325.35 | 60.96 | 51.10 | -462.96 | -1117.24 | 69.01 | 51.10 |
|  | QF | 73.17 | 68.05 | 1169.08 | 80.61 | 64.52 | 259.27 | 75.02 | 61.24 | 249.07 | -851.74 | 80.61 | 61.24 |
| P3 | NF | 75.92 | 69.30 | -1592.84 | 70.86 | 71.10 | -702.42 | 69.71 | 73.75 | -714.74 | 1213.91 | 70.86 | 73.75 |
|  | TF | 81.10 | 71.74 | -1592.84 | 78.60 | 73.44 | -702.42 | 73.85 | 77.24 | -714.74 | 1213.91 | 70.86 | 77.24 |
|  | QF | 90.11 | 101.76 | 300.39 | 146.74 | 90.96 | 262.01 | 125.25 | 91.46 | 347.87 | 1149.73 | 146.74 | 91.46 |
| P4 | NF | 44.24 | 38.11 | -518.79 | 42.78 | 39.23 | 14006.08 | 37.42 | 39.81 | 487.49 | -343.98 | 42.78 | 39.81 |
|  | TF | 55.09 | 46.98 | -518.79 | 53.01 | 47.43 | 14006.08 | 46.91 | 48.17 | 487.49 | -343.98 | 53.01 | 48.17 |
|  | QF | 90.13 | 72.17 | 2799.04 | 78.49 | 72.96 | 168.33 | 74.73 | 71.12 | 148.94 | -411.30 | 78.49 | 71.12 |
| P5 | NF | 60.97 | 51.03 | -691.72 | 59.53 | 53.12 | -306.07 | 56.33 | 53.55 | -280.88 | 1035.03 | 59.53 | 53.55 |
|  | TF | 71.44 | 53.52 | -691.72 | 63.42 | 56.06 | -306.07 | 59.40 | 57.32 | -280.88 | 1035.03 | 63.42 | 57.32 |
|  | QF | 77.89 | 71.17 | 255.72 | 88.57 | 67.39 | 198.97 | 83.27 | 60.80 | 313.34 | 738.28 | 88.57 | 60.80 |
| P6 | NF | 74.96 | 59.57 | -338.12 | 50.03 | 64.37 | -195.99 | 55.23 | 66.92 | -256.93 | 2125.72 | 50.03 | 66.92 |
|  | TF | 106.58 | 73.85 | -338.12 | 63.48 | 81.32 | -195.99 | 68.05 | 86.32 | -256.93 | 2125.72 | 63.48 | 86.32 |
|  | QF | 167.83 | 103.88 | 1429.56 | 102.13 | 116.29 | 3529.74 | 102.11 | 125.08 | -3486.94 | 1699.65 | 102.13 | 125.08 |

CV = coefficient of variation (SD/mean, %), P1-P6 = Pattern1-Pattern6, NF = No Filter, TF = Triplet Filter, QF = Quad Filter

**Table M. Mean within-subject variability of reaction times computed for each Models` each subcategory**

|  | | Model 1 | | Model 2 | | Model 3 | | | Model 4 | | | Model 5 | | | |
| --- | --- | --- | --- | --- | --- | --- | --- | --- | --- | --- | --- | --- | --- | --- | --- |
|  |  | P | R | H | L | HP | HR | LR | H2 | H1 | L | H2P | H1P | H1R | LR |
| SD mean (ms) | NF | 51.38 | 54.72 | 51.64 | 54.34 | 51.38 | 51.09 | 54.34 | 50.77 | 51.76 | 54.34 | 50.77 | 51.39 | 51.09 | 54.34 |
|  | TF | 51.38 | 54.40 | 51.64 | 54.03 | 51.38 | 51.09 | 54.03 | 50.77 | 51.76 | 54.03 | 50.77 | 51.39 | 51.09 | 54.03 |
|  | QF | 49.59 | 52.14 | 49.61 | 52.73 | 49.59 | 48.53 | 52.73 | 48.98 | 49.40 | 52.73 | 48.98 | 49.06 | 48.53 | 52.73 |
| CV mean (%) | NF | 13.67 | 14.28 | 13.80 | 13.97 | 13.67 | 13.88 | 13.97 | 13.42 | 14.03 | 13.97 | 13.42 | 13.89 | 13.88 | 13.97 |
|  | TF | 13.67 | 14.28 | 13.80 | 13.91 | 13.67 | 13.88 | 13.91 | 13.42 | 14.03 | 13.91 | 13.42 | 13.89 | 13.88 | 13.91 |
|  | QF | 13.04 | 13.42 | 13.05 | 13.28 | 13.04 | 12.74 | 13.28 | 12.95 | 12.95 | 13.28 | 12.95 | 12.82 | 12.74 | 13.28 |

SD = Standard Deviaton, CV = Coefficient of Variation (SD/mean, %)

**Table N. Mean within-subject variability (SD) of reaction times computed for each Models’ each subcategory broken down by the ASRT sequences being thought (P1-P6).**

|  | | Model 1 | | Model 2 | | Model 3 | | | Model 4 | | | Model5 | | | |
| --- | --- | --- | --- | --- | --- | --- | --- | --- | --- | --- | --- | --- | --- | --- | --- |
|  |  | P | R | H | L | HP | HR | LR | H2 | H1 | L | H2P | H1P | H1R | LR |
| P1 | NF | 50.01 | 53.48 | 50.23 | 53.03 | 50.01 | 49.59 | 53.03 | 49.43 | 50.17 | 53.03 | 49.43 | 49.87 | 49.59 | 53.03 |
|  | TF | 50.01 | 53.00 | 50.23 | 52.54 | 50.01 | 49.59 | 52.54 | 49.43 | 50.17 | 52.54 | 49.43 | 49.87 | 49.59 | 52.54 |
|  | QF | 48.18 | 50.65 | 48.19 | 51.08 | 48.18 | 47.11 | 51.08 | 48.03 | 47.87 | 51.08 | 48.03 | 47.56 | 47.11 | 51.08 |
| P2 | NF | 51.13 | 55.59 | 51.14 | 55.41 | 51.13 | 50.00 | 55.41 | 50.46 | 51.19 | 55.41 | 50.46 | 51.62 | 50.00 | 55.41 |
|  | TF | 51.13 | 54.82 | 51.14 | 54.76 | 51.13 | 50.00 | 54.76 | 50.46 | 51.19 | 54.76 | 50.46 | 51.62 | 50.00 | 54.76 |
|  | QF | 50.11 | 52.89 | 49.64 | 53.65 | 50.11 | 48.04 | 53.65 | 48.72 | 49.51 | 53.65 | 48.72 | 50.12 | 48.04 | 53.65 |
| P3 | NF | 54.12 | 57.33 | 54.90 | 56.18 | 54.12 | 56.34 | 56.18 | 53.44 | 55.64 | 56.18 | 53.44 | 53.89 | 56.34 | 56.18 |
|  | TF | 54.12 | 57.26 | 54.90 | 55.80 | 54.12 | 56.34 | 55.80 | 53.44 | 55.64 | 55.80 | 53.44 | 53.89 | 56.34 | 55.80 |
|  | QF | 51.68 | 55.20 | 54.72 | 54.11 | 51.68 | 53.48 | 54.11 | 50.58 | 53.19 | 54.11 | 50.58 | 51.60 | 53.48 | 54.11 |
| P4 | NF | 50.56 | 53.78 | 50.61 | 53.95 | 50.56 | 49.13 | 53.95 | 50.13 | 50.44 | 53.95 | 50.13 | 50.64 | 49.13 | 53.95 |
|  | TF | 50.56 | 53.98 | 50.61 | 54.59 | 50.56 | 49.13 | 54.59 | 50.13 | 50.44 | 54.59 | 50.13 | 50.64 | 49.13 | 54.49 |
|  | QF | 48.35 | 50.85 | 47.98 | 53.03 | 48.35 | 45.97 | 23.03 | 47.93 | 47.54 | 53.03 | 47.93 | 47.83 | 45.97 | 53.03 |
| P5 | NF | 51.05 | 53.52 | 51.41 | 53.08 | 51.05 | 51.16 | 53.08 | 50.49 | 51.41 | 53.08 | 50.49 | 50.61 | 51.16 | 53.08 |
|  | TF | 51.05 | 53.13 | 51.41 | 52.60 | 51.05 | 51.16 | 52.60 | 50.49 | 51.41 | 52.60 | 50.49 | 50.61 | 51.16 | 52.60 |
|  | QF | 49.17 | 51.30 | 49.39 | 51.65 | 49.17 | 48.90 | 51.65 | 48.65 | 49.23 | 51.65 | 48.65 | 48.40 | 48.90 | 51.65 |
| P6 | NF | 51.49 | 54.71 | 51.62 | 54.45 | 51.49 | 50.29 | 54.45 | 50.72 | 51.77 | 54.45 | 50.72 | 51.83 | 50.29 | 54.45 |
|  | TF | 51.49 | 54.22 | 51.62 | 53.86 | 51.49 | 50.29 | 53.86 | 50.72 | 51.77 | 53.86 | 50.72 | 51.83 | 50.29 | 53.86 |
|  | QF | 50.28 | 52.05 | 49.94 | 52.93 | 50.28 | 47.74 | 52.93 | 50.24 | 49.18 | 52.93 | 50.24 | 48.97 | 47.74 | 52.93 |

P1-P6 = Pattern1-Pattern6, NF = No Filter, TF = Triplet Filter, QF = Quad Filter

**Table O. Mean within-subject variability (CV) of reaction times computed for each Models’ each subcategory broken down by the ASRT sequences being thought (P1-P6).**

|  | | Model 1 | | Model 2 | | Model 3 | | | Model 4 | | | Model5 | | | |
| --- | --- | --- | --- | --- | --- | --- | --- | --- | --- | --- | --- | --- | --- | --- | --- |
|  | | P | R | H | L | HP | HR | LR | H2 | H1 | L | H2P | H1P | H1R | LR |
| P1 | NF | 13.75 | 14.43 | 13.88 | 14.08 | 13.75 | 13.96 | 14.08 | 13.51 | 14.07 | 14.08 | 13.51 | 13.94 | 13.96 | 14.08 |
|  | TF | 13.75 | 14.40 | 13.88 | 14.00 | 13.75 | 13.96 | 14.00 | 13.51 | 14.07 | 14.00 | 13.51 | 13.94 | 13.96 | 14.00 |
|  | QF | 13.06 | 13.52 | 13.08 | 13.33 | 13.06 | 12.81 | 13.33 | 13.04 | 12.98 | 13.33 | 13.04 | 12.85 | 12.81 | 13.33 |
| P2 | NF | 13.63 | 14.38 | 13.69 | 14.07 | 13.63 | 13.60 | 14.07 | 13.38 | 13.88 | 14.07 | 13.38 | 13.93 | 13.60 | 14.07 |
|  | TF | 13.63 | 14.31 | 13.69 | 13.97 | 13.63 | 13.60 | 13.97 | 13.38 | 13.88 | 13.97 | 13.38 | 13.93 | 13.60 | 13.97 |
|  | QF | 13.29 | 13.61 | 13.17 | 13.34 | 13.29 | 12.73 | 13.34 | 13.04 | 13.06 | 13.34 | 13.04 | 13.15 | 12.73 | 13.34 |
| P3 | NF | 14.06 | 14.67 | 14.32 | 14.20 | 14.06 | 14.92 | 14.20 | 13.76 | 14.77 | 14.20 | 13.76 | 14.33 | 14.92 | 14.20 |
|  | TF | 14.06 | 14.71 | 14.32 | 14.09 | 14.06 | 14.92 | 14.09 | 13.76 | 14.77 | 14.09 | 13.76 | 14.33 | 14.92 | 14.09 |
|  | QF | 13.32 | 13.87 | 13.55 | 13.37 | 13.32 | 13.63 | 13.37 | 13.03 | 13.63 | 13.37 | 13.03 | 13.28 | 13.63 | 13.37 |
| P4 | NF | 13.17 | 13.73 | 13.24 | 13.58 | 13.17 | 13.06 | 13.58 | 13.01 | 13.33 | 13.58 | 13.01 | 13.30 | 13.06 | 13.58 |
|  | TF | 13.17 | 13.85 | 13.24 | 13.75 | 13.17 | 13.06 | 13.75 | 13.01 | 13.33 | 13.75 | 13.01 | 13.30 | 13.06 | 13.75 |
|  | QF | 12.40 | 12.79 | 12.32 | 13.05 | 12.40 | 11.80 | 13.05 | 12.42 | 12.14 | 13.05 | 12.42 | 12.13 | 11.80 | 13.05 |
| P5 | NF | 13.54 | 14.01 | 13.70 | 13.71 | 13.54 | 13.89 | 13.71 | 13.28 | 13.95 | 13.71 | 13.28 | 13.71 | 13.89 | 13.71 |
|  | TF | 13.54 | 13.99 | 13.70 | 13.62 | 13.54 | 13.89 | 13.62 | 13.28 | 13.95 | 13.62 | 13.28 | 13.71 | 13.89 | 13.62 |
|  | QF | 12.93 | 13.24 | 12.97 | 13.13 | 12.93 | 12.78 | 13.13 | 12.85 | 12.89 | 13.13 | 12.85 | 12.67 | 12.78 | 13.13 |
| P6 | NF | 13.92 | 14.50 | 14.02 | 14.22 | 13.92 | 13.89 | 14.22 | 13.64 | 14.25 | 14.22 | 13.64 | 14.19 | 13.89 | 14.22 |
|  | TF | 13.92 | 14.43 | 14.02 | 14.05 | 13.92 | 13.89 | 14.05 | 13.64 | 14.25 | 14.05 | 13.64 | 14.19 | 13.89 | 14.05 |
|  | QF | 13.36 | 13.57 | 13.29 | 13.51 | 13.36 | 12.72 | 13.51 | 13.39 | 13.06 | 13.51 | 13.39 | 12.94 | 12.72 | 13.51 |

P1-P6 = Pattern1-Pattern6, NF = No Filter, TF = Triplet Filter, QF = Quad Filter


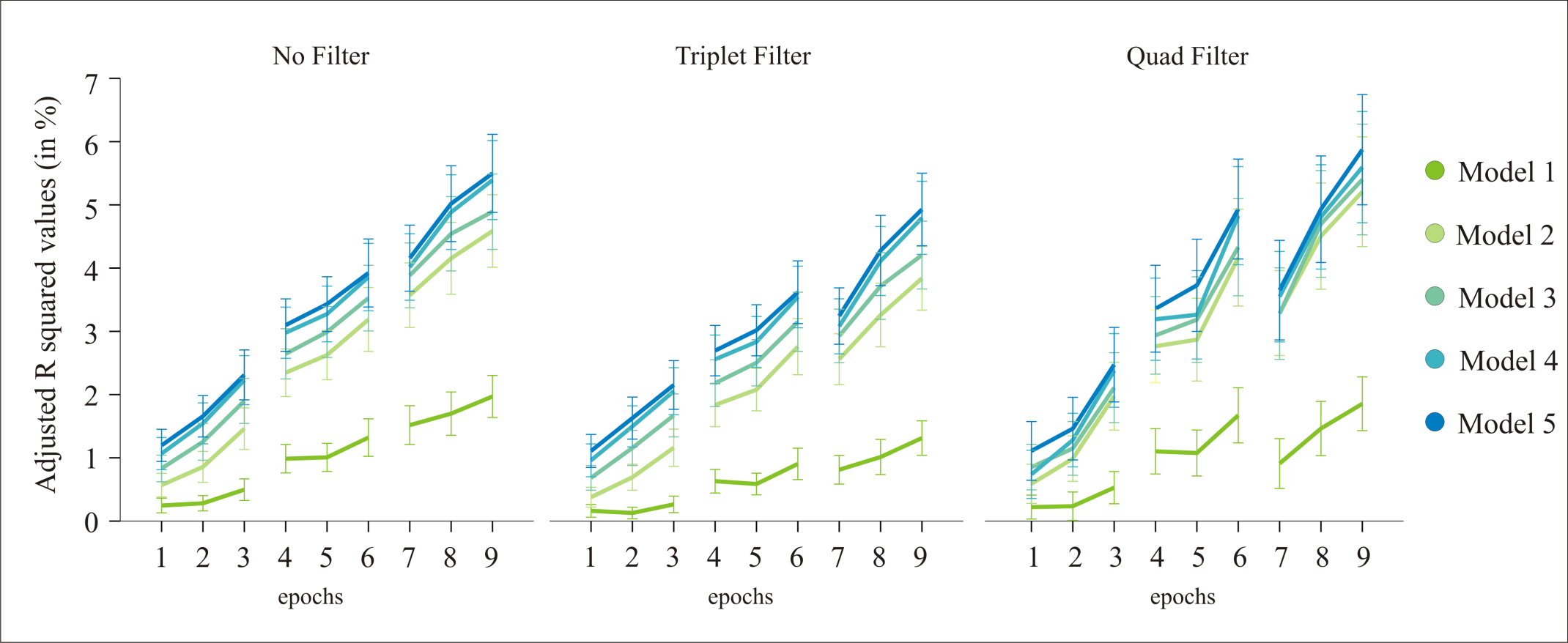


**Figure A**

**
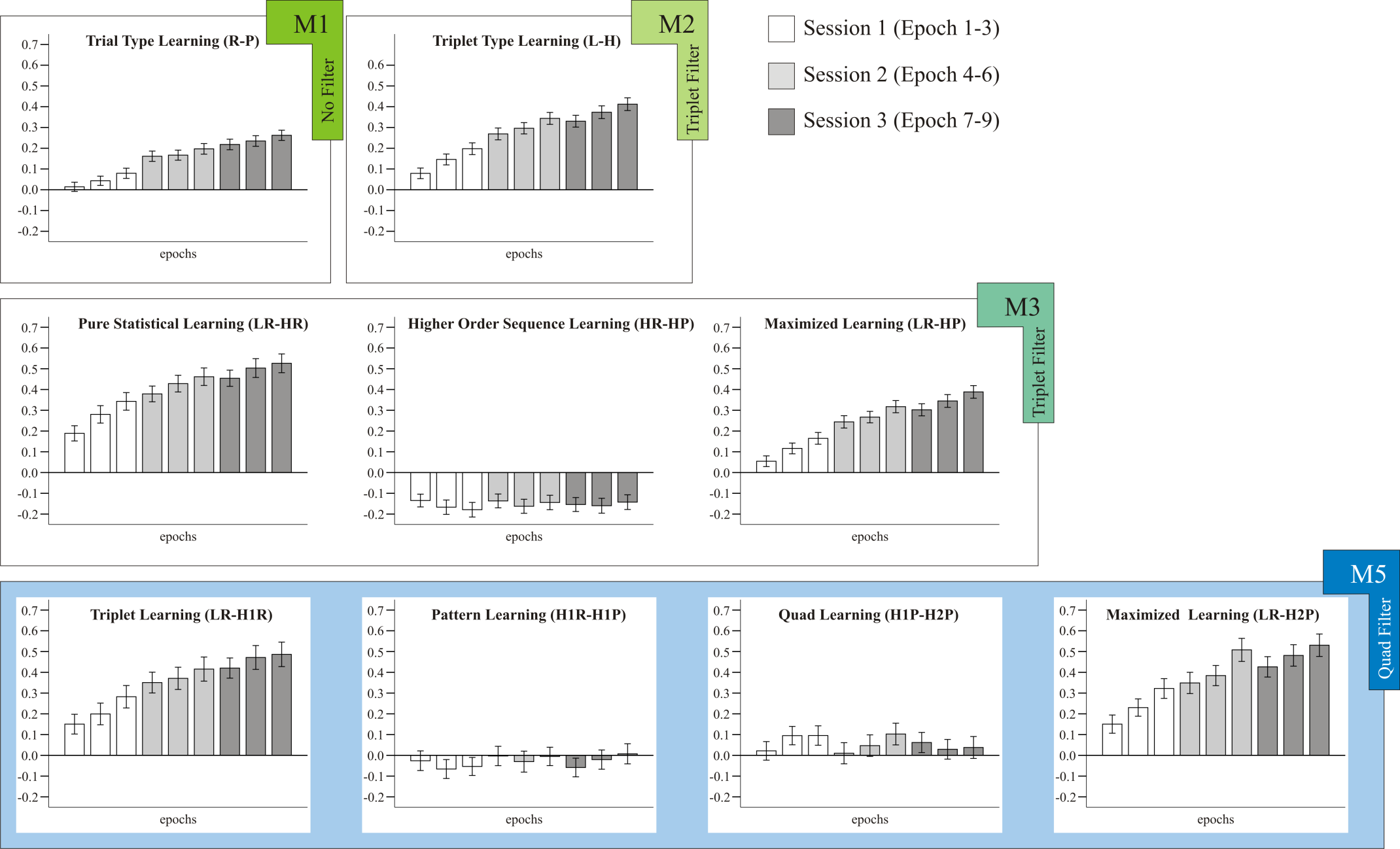
**

**Figure B**

**
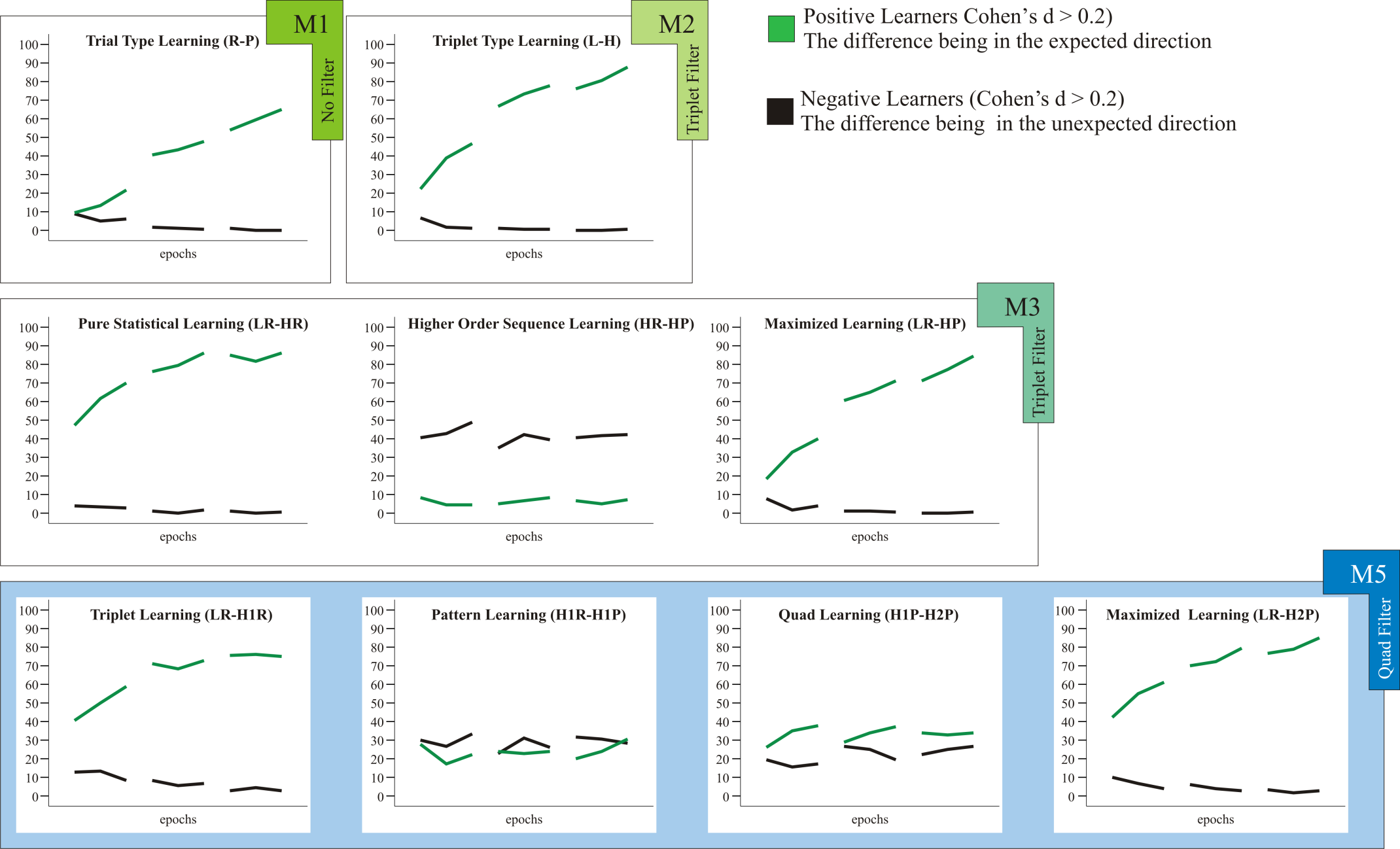
**

**Figure C**


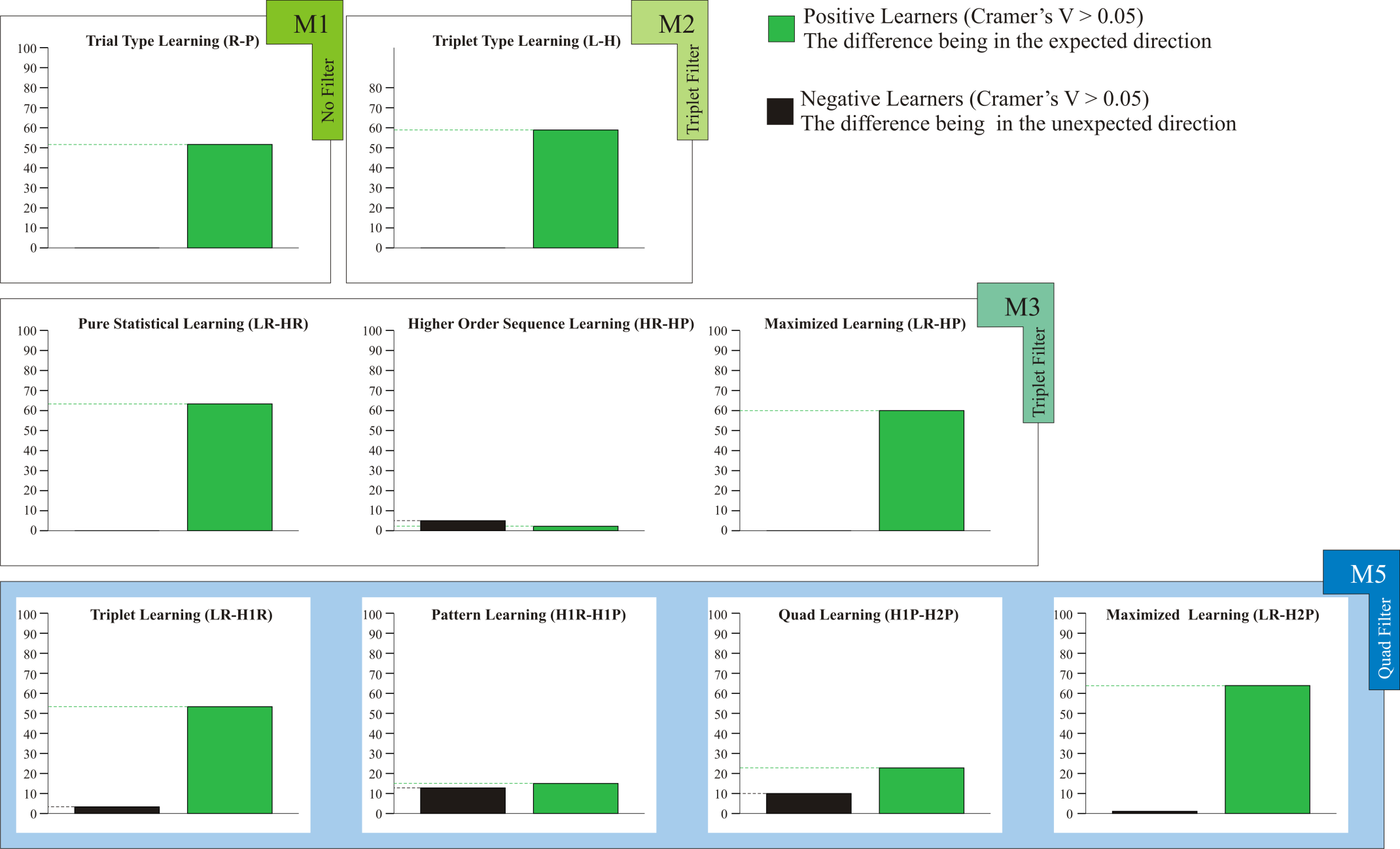


**Figure D**

**Table P. Correspondance between the „reliably positive learner” status of participants with the currently proposed method vs. the usual analysis methods.** Phi coefficients were calculated and are shown in the table. The scores are based on reaction times.

|  | | M1  No Filter | M2  Triplet Filter | M3  Triplet Filter | | |
| --- | --- | --- | --- | --- | --- | --- |
|  |  | **R-P**  *TrialType effect* | **L-H**  *Seq. Spec. L.* | **LR-HR**  *Pure Stat. L.* | **HR-HP**  *Higher Ord. L.* | **LR-HP**  *Max. Learning* |
| M5  Quad  Filter | **LR-H1R**  *Triplet Learning* | .301** | .389** | .442** | N/A | .442** |
|  | **H1R-H1P** *Pattern Learning* | .141 | -.092 | -.243** | N/A | -.031 |
|  | **H1P-H2P**  *Quad Learning* | -.100 | -.010 | -.087 | N/A | -.064 |
|  | **LR-H2P**  *Max. Learning* | .242** | .392** | .257** | N/A | .293** |

M1-M5: Model1 – Model 5

N/A – statistics not available due to the lack of reliable *M3 Higher Order Learning* learners

** significant, p < .005
